# Quantitative Relationship Between White Matter Hyperintensity Volume and Fazekas Score on Brain MRI

**DOI:** 10.1161/STROKEAHA.125.055101

**Published:** 2026-07-15

**Authors:** Yu-Yuan Xu, Francesca M. Chappell, Maria Valdes Hernandez, Carmen Arteaga Reyes, Una Clancy, Daniela Jaime Garcia, Stephen Makin, Michael Stringer, Michael Thrippleton, Stewart Wiseman, Yajun Cheng, Dillys Xiaodi Liu, Rosalind Brown, Fergus Doubal, Ian J. Deary, Simon Cox, Danielle Kerkhofs, Julie Staals, Walter Backes, Robert van Oostenbrugge, Anna Kopczak, Marco Duering, Joanna M. Wardlaw

**Affiliations:** China National Clinical Research Center for Neurological Diseases, Beijing Tiantan Hospital, Capital Medical University (Y.-Y.X.).; Institute for Neuroscience and Cardiovascular Research (Y.-Y.X., F.M.C., M.V.H., C.A.R., U.C., D.J.G., S.M., M.S., M.T., S.W., R.B., F.D., J.M.W.), The University of Edinburgh, United Kingdom.; UK Dementia Research Institute (Y.-Y.X., F.M.C., M.V.H., C.A.R., U.C., D.J.G., S.M., M.S., M.T., S.W., R.B., F.D., J.M.W.), The University of Edinburgh, United Kingdom.; Department of Psychology (I.J.D., S.C.), The University of Edinburgh, United Kingdom.; Centre for Rural Health, University of Aberdeen, United Kingdom (S.M.).; Department of Neurology, West China Hospital, Sichuan University, Chengdu (Y.C.).; Department of Psychiatry and Behavioral Sciences, University of California, San Francisco (D.X.L.).; Department of Neurology, CARIM School for Cardiovascular Diseases, Maastricht University Medical Center, the Netherlands (D.K., J.S., W.B., R.v.O.).; Institute for Stroke and Dementia Research, Ludwig-Maximilians Universität, Munich, Germany (A.K., M.D.).; Medical Image Analysis Center (MIAC AG) and Department of Biomedical Engineering, University of Basel, Switzerland (M.D.).

**Keywords:** cerebral small vessel diseases, image processing, computer-assisted, magnetic resonance imaging, reproducibility of results, white matter

## Abstract

**BACKGROUND:**

The Fazekas score is widely used to grade white matter hyperintensities (WMHs) in cerebral small vessel disease, yet the equivalent volume of each grade is unclear. We quantified the correspondence between Fazekas scores and WMH volume, normalized ratios, and derived conversion equations across populations.

**METHODS:**

We analyzed 1220 participants from 4 mostly United Kingdom–based cohorts representing different cerebral small vessel disease severities: community-dwelling (LBC1936 [Lothian Birth Cohort 1936]), stroke (MSS2 [Mild Stroke Study 2] and MSS3 [Mild Stroke Study 3]), and a cerebral autosomal dominant arteriopathy with subcortical infarcts and leukoencephalopathy-enriched cohort (INVESTIGATE [Imaging Neuro-Vascular, Endothelial and Structural Integrity in Preparation to Treat Small Vessel Diseases]). WMH burden was quantified as absolute WMH volume and as normalized ratios: WMH volume as a percentage of brain volume and WMH volume as a percentage of intracranial volume. Transition zones were defined as the overlap of WMH volume interquartile ranges between adjacent Fazekas scores. Linear, exponential (nonlinear least squares), and log-linear (ln WMH≈Fazekas score and zeros excluded) models were fitted; model fit was assessed using mean squared error, (pseudo-)R^2^, and Vuong tests. Periventricular-versus-deep WMH patterns within each total Fazekas score were compared using the Kruskal-Wallis test.

**RESULTS:**

The pooled cohort had a mean age of 69.4±8.9 years; 700 of 1227 records (57.1%) were male. WMH burden increased monotonically with a Fazekas score in all cohorts. Adjacent-grade separation was strongest in LBC1936 (all *P*≤0.002) but weaker at lower grades in MSS2, MSS3, and INVESTIGATE. The widest transition zones occurred between Fazekas grades of 3 to 4 and 4 to 5, whereas a clear WMH volume gap separated grades 5 and 6. Exponential models generally fit better than linear models, with lower mean squared error and supportive Vuong tests in most cohorts. Log-linear sensitivity analyses showed similar fitted trajectories after back-transformation. WMH burden did not differ consistently by periventricular-versus-deep distribution within Fazekas grades.

**CONCLUSIONS:**

WMH burden followed an exponential trajectory across Fazekas scores, with population-dependent progression. The resulting conversion equations enabled bidirectional translation between visual Fazekas score and quantitative WMH volume, facilitating cross-study comparison, large-scale epidemiology, and might facilitate clinical decision-making in cerebral small vessel disease.

White matter hyperintensities (WMHs) are a hallmark feature of cerebral small vessel disease (CSVD), visualized as hyperintense regions on T2‐weighted and fluid-attenuated inversion recovery (FLAIR) magnetic resonance imaging (MRI) sequences, particularly in the periventricular and deep white matter.^1,2^ Numerous qualitative visual rating scales have been developed for WMH assessment,^1,3^ with the Fazekas score^4^ being the most commonly used and validated method for evaluating WMH severity. However, in recent years, many automated computational methods and deep learning–based algorithms have emerged to quantify WMH volume^5,6^ although these have yet to be used routinely in clinical practice where a visual estimation remains common.

Both qualitative and quantitative approaches have their advantages and disadvantages.^7^ There have been several previous comparisons of visual rating scales and quantitative WMH volume measurements,^8–11^ but their results differed in sources of disagreement, sample size, and study population. Although quantitative WMH volumetry is often regarded as superior due to its objectivity, reproducibility, and suitability for statistical analysis,^12^ it did not outperform the simple, visually based rating scales in terms of the relationships with clinical outcomes, such as physical performance and cognition.^13^ Moreover, of recent papers describing normative WMH volumes (normalized for intracranial volume [ICV] or brain volume) in population studies across different ages,^14,15^ none of them have directly equated WMH volume with Fazekas scores.

The aim of this study is to examine the association between Fazekas scores and WMH volume (using raw volume or normalized ratios) and to determine whether this relationship varies by clinical presentation or CSVD severity, using several data sets representing different severities of CSVD across different populations. We also aim to develop a simple conversion equation to translate Fazekas scores into WMH volumes (and vice versa) and to explore how the periventricular WMH (PVWMH) and deep WMH (DWMH) components individually contribute to the overall WMH burden.

Such insights could be valuable in both epidemiological research and clinical practice by enhancing risk stratification and standardizing WMH assessment while also shedding light on patterns of WMH formation.

## Methods

### Data Availability Statement

The data that support the findings of this study are available from the corresponding author upon reasonable request, subject to appropriate institutional and ethical approvals.

### Reporting Guideline

This study is reported according to the Strengthening the Reporting of Observational Studies in Epidemiology guideline.

### Study Participants

We identified participants from 4 observational cohort studies conducted in the United Kingdom. LBC1936 (Lothian Birth Cohort 1936)^16^ is a longitudinal study of community-dwelling older adults born in 1936 (N=1091) with the main aim to study nonpathological cognitive aging in later life. We extracted data from Wave 2 of LBC1936, at which the first brain MRI was obtained between 2007 and 2010. The MSS2 (Mild Stroke Study 2; from 2010 to 2013; N=264)^17^ and MSS3 (Mild Stroke Study 3; from 2018 to 2021; N=230)^18^ recruited patients with lacunar or mild cortical ischemic stroke presenting to Edinburgh/Lothian stroke services. For the INVESTIGATE study (Imaging Neuro-Vascular, Endothelial and Structural Integrity in Preparation to Treat Small Vessel Diseases),^19^ we assessed patients recruited in Edinburgh, Munich, and Maastricht from 2017 to 2019 (N=77).

Participants were excluded if they did not undergo MRI scanning or had missing brain or WMH volume measurements. Participants coenrolled in >1 cohort (paired study IDs listed in Table S1) were retained in each relevant cohort for cohort-specific analyses but counted once in analyses of unique individuals.

Cohorts were analyzed separately as the primary analytic strata because recruitment context and MRI acquisition protocols differed across studies and to retain discrete cohort participant characteristics. For clinical interpretability, we additionally present descriptive summaries after grouping cohorts as community-dwelling (LBC1936), stroke (MSS2 and MSS3 combined, plus lacunar stroke participants from INVESTIGATE), and cerebral autosomal dominant arteriopathy with subcortical infarcts and leukoencephalopathy (CADASIL; confirmed CADASIL drawn from the INVESTIGATE cohort). These community/stroke/CADASIL groupings are used for descriptive comparisons only and do not replace the cohort-stratified modeling.

### Standard Protocol Approvals, Registrations, and Patient Consents

Ethical permission for the LBC1936 study was obtained from the Multi-Center Research Ethics Committee for Scotland (Wave 1: MREC/01/0/56), the Lothian Research Ethics Committee (Wave 1: LREC/2003/2/29), and the Scotland A Research Ethics Committee (Waves 2, 3, 4, and 5: 07/MRE00/58). The MSS2 study was approved by the Lothian Research Ethics Committee (ref 09/S1101/54). MSS3 and INVESTIGATE received approval from the Southeast Scotland Regional Ethics Committee (ref 18/SS/0044 and 16/SS/0123, respectively). All participants provided written informed consent.

All procedures performed in studies involving human participants were in accordance with the ethical standards of the national research ethics committee and with the 1964 Helsinki Declaration and its later amendments or comparable ethical standards.

### Imaging Acquisition

For the current analysis, we included available participants with MRI data at baseline for MSS2, MSS3, and INVESTIGATE and the Wave 2 images of LBC1936. In the LBC1936 study, Wave 1 data (no MRI) were acquired between 2004 and 2007. At Wave 2, from 2007 to 2010, 724 participants underwent structural brain MRI on a 1.5T GE Signa Horizon HDx clinical scanner (General Electric, Milwaukee, WI).^20^ The study protocols included diffusion-weighted imaging, 2-dimensional T2-weighted, FLAIR, fast spoiled gradient-echo, and 3-dimensional T1-weighted sequences and are published elsewhere.^16^ In the MSS2 study, participants underwent a diagnostic MRI at presentation and a repeat MRI at baseline (within 3 months of index stroke ±1 week) and 1 year, all on the same 1.5T scanner as for the LBC1936. The imaging protocols include diffusion-weighted imaging, 2-dimensional T2-weighted, FLAIR, gradient-recalled echo, and 3-dimensional T1-weighted sequences (as for LBC1936) and are published elsewhere.^17,21^ In the MSS3 study, participants underwent baseline (within 3 months of index stroke ±1 week) MRI on a 3T scanner (Prisma, Siemens Healthcare, Erlangen, Germany) with 3-dimensional isotropic T1-weighted, 3-dimensional T2-weighted, 3-dimensional FLAIR, diffusion-weighted imaging, and susceptibility-weighted imaging sequences (full details described previously).^18^ In the INVESTIGATE study, participants had a research MRI using the same 3T scanner and sequences as described for MSS3 in Edinburgh,^19^ the same 3T Siemens model in Munich, and a Philips 3T in Maastricht; full harmonization details are published.

### Imaging Processing and Analysis

WMHs on structural MRI sequences were identified according to the STRIVE criteria (Standards for Reporting Vascular Changes on Neuroimaging)^1^ and graded by different trained raters in each study using the Fazekas validated qualitative scales for WMH^4^; a total score ranging from 0 to 6 was obtained by summing the PVWMH (0–3) and DWMH (0–3) Fazekas scores. A board-certified neuroradiologist (the same for all 4 cohorts) checked and confirmed all the scores.

All computational image analyses were performed for all cohorts by the same team using the same basic methods. All sequences were coregistered to the T2-weighted image using the linear image registration tool (FLIRT)^22^ from the FMRIB software library (FSL; FSL FLIRT).^23^ ICVs were automatically generated from the coregistered proton density image (or equivalent contrast spoiled gradient echo with flip angle=2° acquired as part of the QT1 acquisition if proton density acquisition was not available) using the brain extraction tool.^24^ All ICV masks were visually checked and manually edited when necessary.

Brain volume was estimated using a hybrid approach that combined the outputs from FSL FAST (FSL automated segmentation tool) and Freesurfer (https://surfer.nmr.mgh.harvard.edu/), with both run using the manually corrected ICV. WMH candidate regions were derived on the T2-registered FLAIR images by first creating tissue masks via hierarchically thresholding T2-registered FLAIR images and then reducing predictable false positives adjacent to the choroid plexus, aqueduct, and third and fourth ventricles using Freesurfer-derived anatomic information. Hyperintense voxels were identified by thresholding FLAIR intensities to >1.69 SDs above the mean brain-tissue intensity. To exclude hyperintensities unlikely to reflect pathology, a lesion distribution probabilistic template was applied to the threshold images.^25^ Further refinement was achieved by applying Gaussian smoothing, followed by removing voxels with an intensity *Z* score <0.95.

The resulting WMH binary masks were visually inspected and manually corrected for artifact-related false positives that might have been missed by the automatic pipeline. Manual mask correction was performed blinded to clinical data. This workflow has been validated in older people with SVD and mild stroke.^7,17^ Finally, old and acute stroke lesions were manually delineated on FLAIR by an experienced rater, guided by other sequences including diffusion-weighted imaging. Stroke lesion masks were reviewed with a neuroradiologist and revised if needed. Before final WMH volume extraction, all identified stroke lesions were excluded from WMH masks and WMH volume estimates to ensure accurate WMH quantification.

Normalized WMH ratios were expressed as percentages in 2 forms: WMH volume as a percentage of brain volume (WMH%Brain)=WMH volume/brain volume×100% and WMH volume as a percentage of ICV (WMH%ICV)=WMH volume/ICV×100%; both percentage measures were subsequently log-transformed for modeling. For each Fazekas score, we calculated the interquartile range (IQR, Q1–Q3) of WMH volume, WMH%Brain, and WMH%ICV. For every pair of adjacent Fazekas scores (0–1, 1–2, …, 5–6), we defined the transition zone as the overlap of their 2 IQRs, if any. If the IQRs did not overlap, we defined the gap as the interval between the upper quartile (Q3) of the lower Fazekas grade and the lower quartile (Q1) of the higher grade.

### Statistical Analysis

We performed all statistical analyses with R, version 4.3.0. Categorical variables were expressed as absolute numbers with percentages, while continuous variables were reported as mean±SD or median with IQR, as appropriate. Comparisons among the total sample and 4 study groups were conducted using the ANOVA test for continuous variables and the χ^2^ test for categorical variables. ANOVA was used for descriptive baseline comparisons only; the main analyses of WMH burden relied on medians and IQRs, nonparametric tests, and model-based approaches.

We presented WMH volume and normalized ratios for each Fazekas score as mean±SD and median (IQR) in the total sample and 4 cohorts. Differences in baseline WMH volume and normalized ratios across cohorts were evaluated using ANOVA, and pairwise differences between consecutive Fazekas scores were analyzed using the Mann-Whitney *U* test. The median (IQR) of WMH volume and normalized ratios in the community-dwelling, stroke, and CADASIL groups were also presented.

We applied both linear and exponential models to describe the relationship between Fazekas scores (ranging from 0 to 6) and WMH burden, including total WMH volume and normalized ratios (WMH%Brain and WMH%ICV) in the total sample and 4 cohorts. The exponential model was fitted using nonlinear least squares (NLS) directly on the original WMH volume. To compare the fit of linear and exponential models, we reported mean squared error and R^2^ values, and used the Vuong test to assess model performance.^26^

In secondary analyses, we also fitted linear models on the natural log-transformed (ln) WMH volume and normalized ratios. Participants with WMH volume = 0 mL (N=18; 16 from LBC1936 and 2 from MSS2) were excluded from the log-transformed models to avoid undefined log(0) values. We then reestimated (1) an exponential model using NLS on the raw (nontransformed) data and (2) a log-linear model using ordinary least squares on the ln-transformed values as a sensitivity analysis. A pseudo-R^2^ on the raw data and an R^2^ on the log data were calculated.

Finally, we used the Kruskal-Wallis test with post hoc pairwise comparisons to determine whether different PVWMH and DWMH combination groups within a given total Fazekas score had significantly different WMH volumes.

No formal correction for multiple testing was applied because many comparisons were descriptive or exploratory and involved correlated WMH measures across related Fazekas strata and cohorts; therefore, results were interpreted based on the consistency and magnitude of patterns across analyses rather than isolated nominal *P* values.

## Results

### Study Population

**Figure 1.**
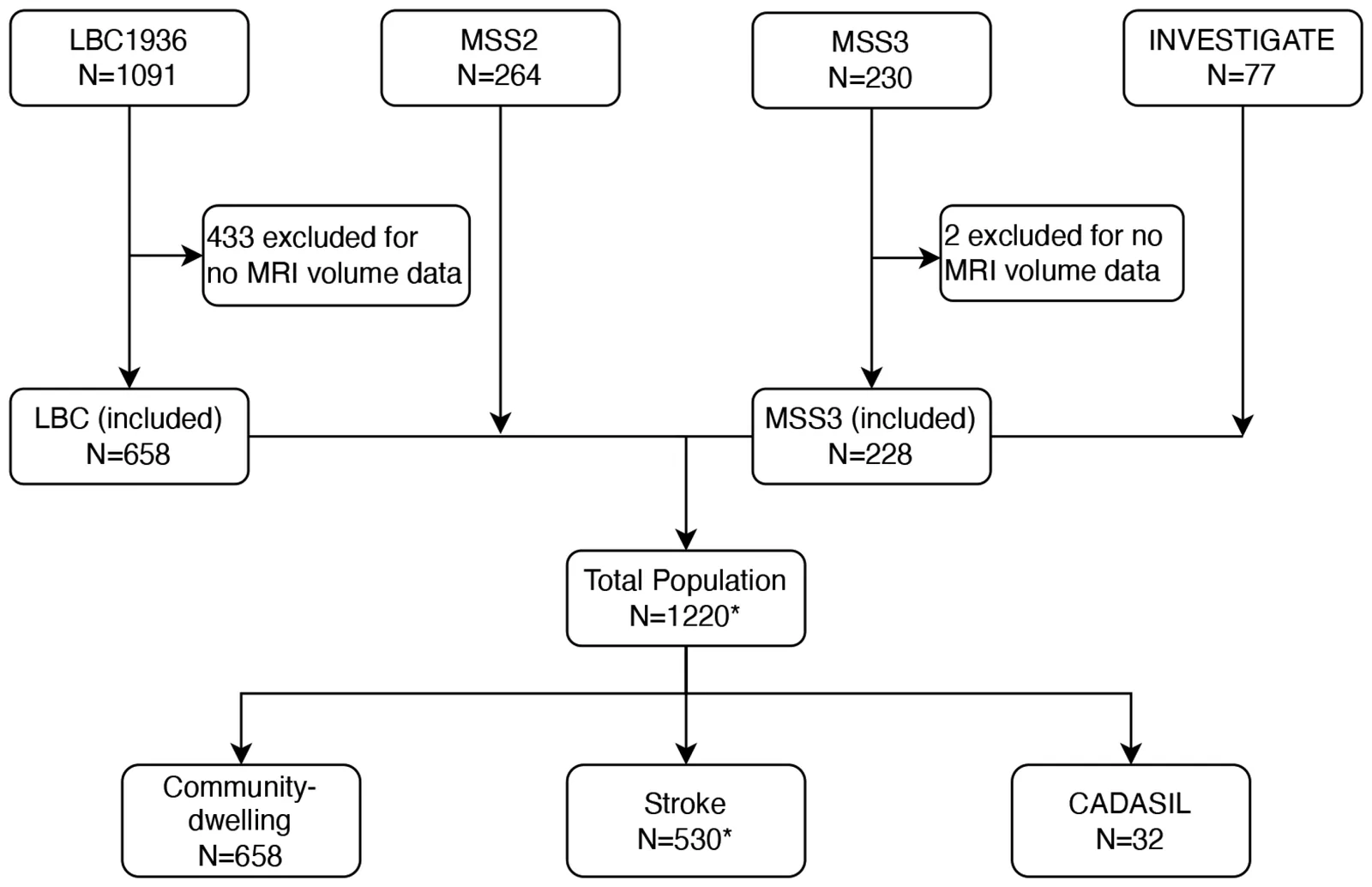
**Flow diagram of data composition and participants enrolled and analyzed.** Note that 7 stroke participants were coenrolled in both MSS3 (Mild Stroke Study 3) and INVESTIGATE (Imaging Neuro-Vascular, Endothelial and Structural Integrity in Preparation to Treat Small Vessel Diseases; paired study IDs are listed in Table S1). Thus, the data set contains 1227 analytic records (537 stroke) but corresponds to 1220 individuals (530 with stroke). CADASIL indicates cerebral autosomal dominant arteriopathy with subcortical infarcts and leukoencephalopathy; LBC1936, Lothian Birth Cohort 1936. MRI, magnetic resonance imaging; MSS2, Mild Stroke Study 2; and WMH, white matter hyperintensity.

After exclusions, the analytic data set comprised 1227 records from 1220 individuals across 4 cohorts: 658 from LBC1936, 264 from MSS2, 228 from MSS3, and 77 from INVESTIGATE (flowchart, Figure 1). Seven participants were coenrolled in MSS3 and INVESTIGATE and, therefore, account for the difference between record-based and unique-individual totals (paired study IDs listed in Table S1).

In the total sample, the mean age was 69.37±8.86 years, 700 participants (57.05%) were men, and the mean WMH volume was 16.90±22.66 mL. Across cohorts, WMH burden was lowest in LBC1936, intermediate in MSS2 and MSS3, and highest in the INVESTIGATE cohort, consistent with its enrichment for CADASIL cases (Table 1). The distribution of Fazekas scores differed across cohorts, with LBC1936 concentrated mainly at lower-to-intermediate scores and INVESTIGATE showing a higher proportion of severe scores (Figure S1).

**Table 1.**
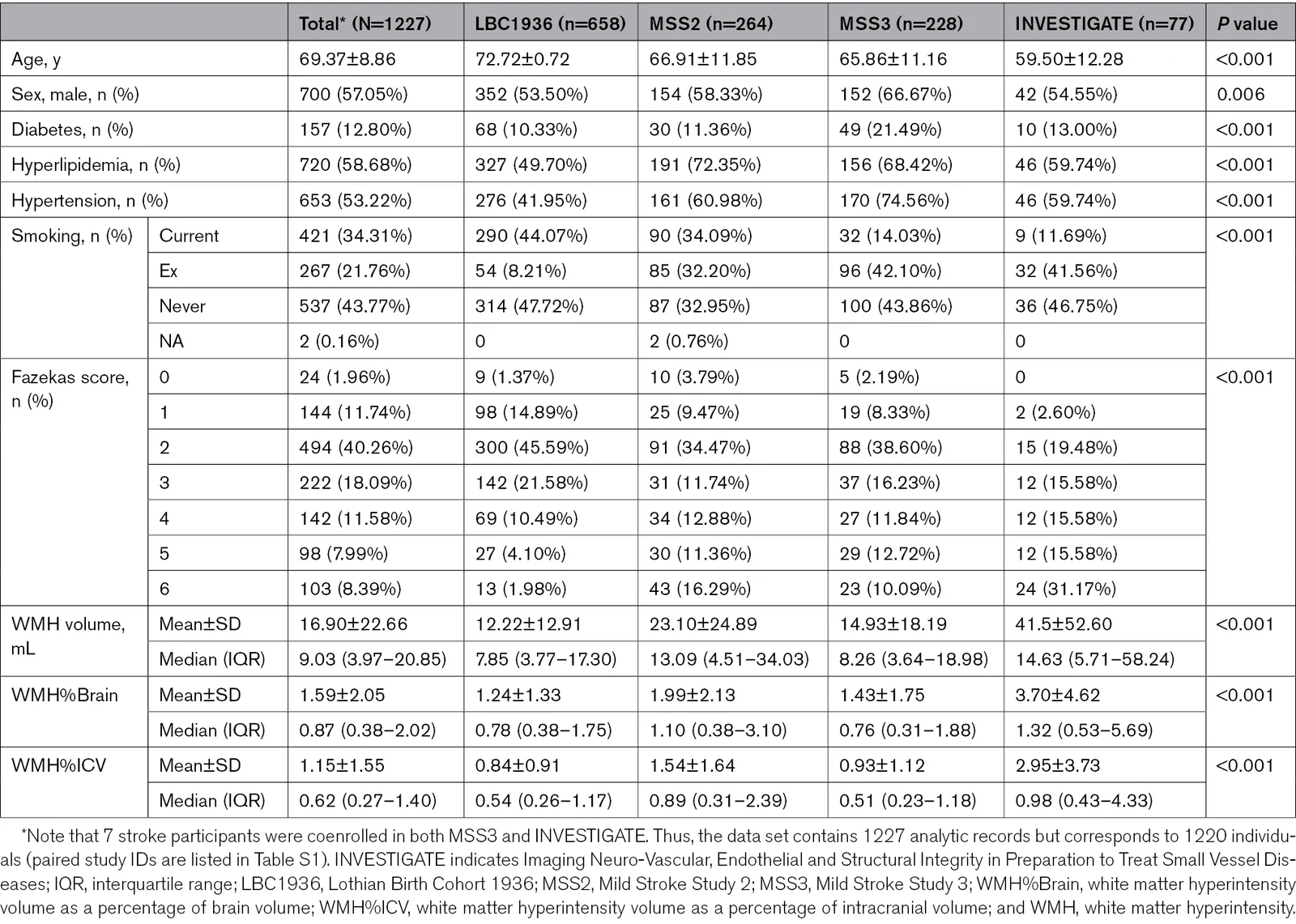
Baseline Characteristics of the Study Population

### WMH Volume, WMH%Brain, and WMH%ICV Across Fazekas Scores

Across the total sample and within each cohort, WMH volume, WMH%Brain, and WMH%ICV increased monotonically with higher Fazekas score (Table S2). The INVESTIGATE cohort consistently exhibited a higher WMH burden than the other 3 groups, especially at higher Fazekas scores.

For clinical interpretability, we additionally summarized WMH burden by mutually exclusive clinical categories (community, stroke, and CADASIL; Table S3). In this presentation, stroke participants had higher WMH burden than community participants at Fazekas score of 0 to 1; at intermediate grades (Fazekas score, 2–5), the community group showed higher median burdens and percentages, consistent with the older age distribution in the community cohort (Table 1); and the 2 groups converged again at Fazekas score of 6. CADASIL cases appeared only at Fazekas score ≥3 and showed the highest median burdens across grades.

### Adjacent-Grade Separation and Transition Zones

**Figure 2.**
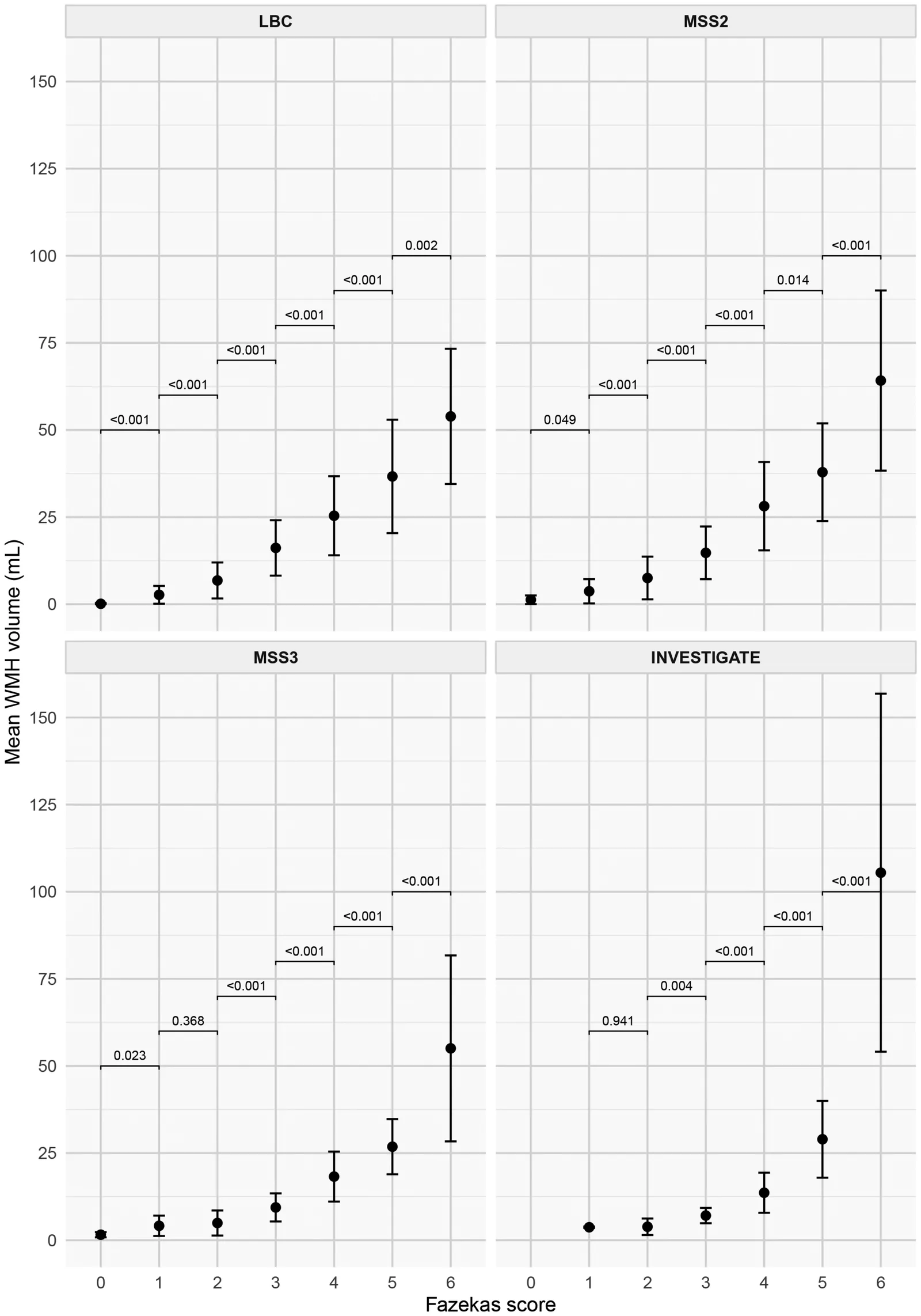
**White matter hyperintensity (WMH) volume differences between adjacent Fazekas scores across LBC1936 (Lothian Birth Cohort 1936), MSS2 (Mild Stroke Study 2), MSS3 (Mild Stroke Study 3), and INVESTIGATE (Imaging Neuro-Vascular, Endothelial and Structural Integrity in Preparation to Treat Small Vessel Diseases).** Grouped point plot with error bars showing differences in WMH volume between each pair of adjacent Fazekas scores across 4 cohorts. The *x* axis displayed the Fazekas scores, and the *y* axis represented the mean WMH volume, with error bars indicating the SD for each cohort. Pairwise *P* values comparing adjacent Fazekas scores were annotated on the plot.

Adjacent-grade separation based on WMH volume is shown in Figure 2, with corresponding *P* values provided in Table S4. In LBC1936, mean WMH volume increased stepwise with significant differences between all adjacent Fazekas grades (all *P*≤0.002; most *P*<0.001). In MSS2, adjacent-grade differences were also generally significant although separation was weaker at the lowest and mid-high grades (0–1, *P*=0.049; 4–5, *P*=0.014). In MSS3, discrimination was limited at the lowest grades (0–1, *P*=0.023; 1–2, *P*=0.368) but became robust from Fazekas score of 2 onward (all *P*<0.001 for 2–3 through 5–6). In INVESTIGATE, adjacent-grade separation was minimal at Fazekas score of 0 to 1 (*P*=0.941) and modest at 2 to 3 (*P*=0.004) but strong from Fazekas score of 3 onward (all *P*<0.001 for 3–4 through 5–6).

Transition-zone analyses (overlap of IQRs) indicated that the greatest overlap occurred between Fazekas scores of 3 to 4 (WMH volume, 15.1–19.0 mL) and 4 to 5 (WMH volume, 24.8–31.6 mL) across measures, whereas a distinct gap was observed between Fazekas scores of 5 and 6, suggesting better discrimination at the severe end of the scale (Figure S2).

### Linear and Exponential Equations of WMH Volume, WMH%Brain, and WMH%ICV Across Fazekas Score

**Figure 3.**
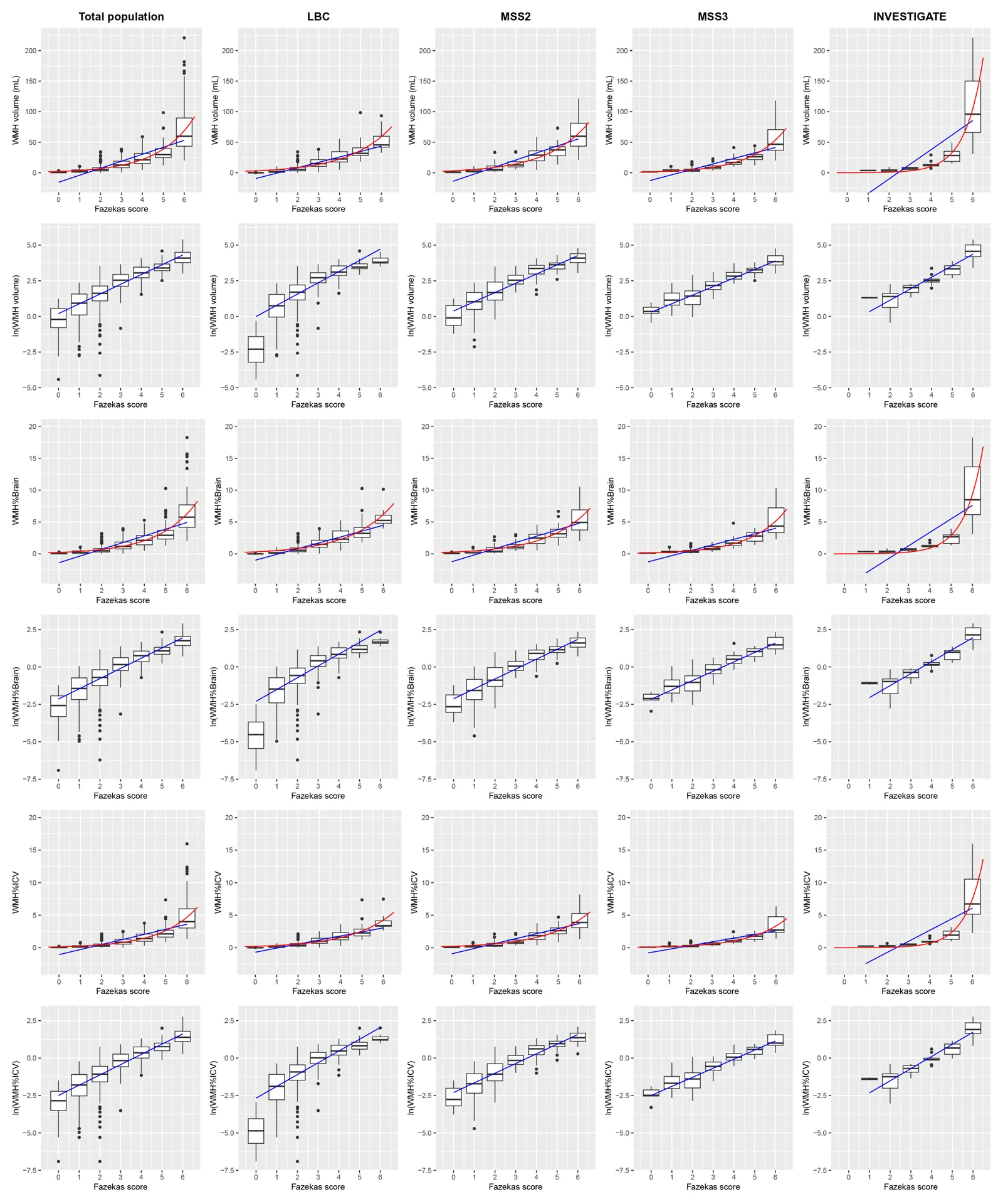
**Linear (blue), exponential (red), and log-linear models with box plots for the white matter hyperintensity (WMH) volume and normalized ratios across Fazekas scores in total population, LBC1936 (Lothian Birth Cohort 1936), MSS2 (Mild Stroke Study 2), MSS3 (Mild Stroke Study 3), and INVESTIGATE (Imaging Neuro-Vascular, Endothelial and Structural Integrity in Preparation to Treat Small Vessel Diseases).** ICV indicates intracranial volume.

Table 2 presents the fitted equations for each model, while the main cohort-level patterns were summarized below. Both linear and exponential models showed increasing WMH burden with a higher Fazekas score. Exponential models showed similar growth-rate parameters across the 3 WMH measures within each cohort. The estimated exponential slopes were comparable in LBC1936 and MSS2 (≈0.49–0.50), modestly higher in MSS3 (≈0.59–0.60), and substantially higher in INVESTIGATE (≈1.17–1.19; Table 2; Figure 3).

**Table 2.**
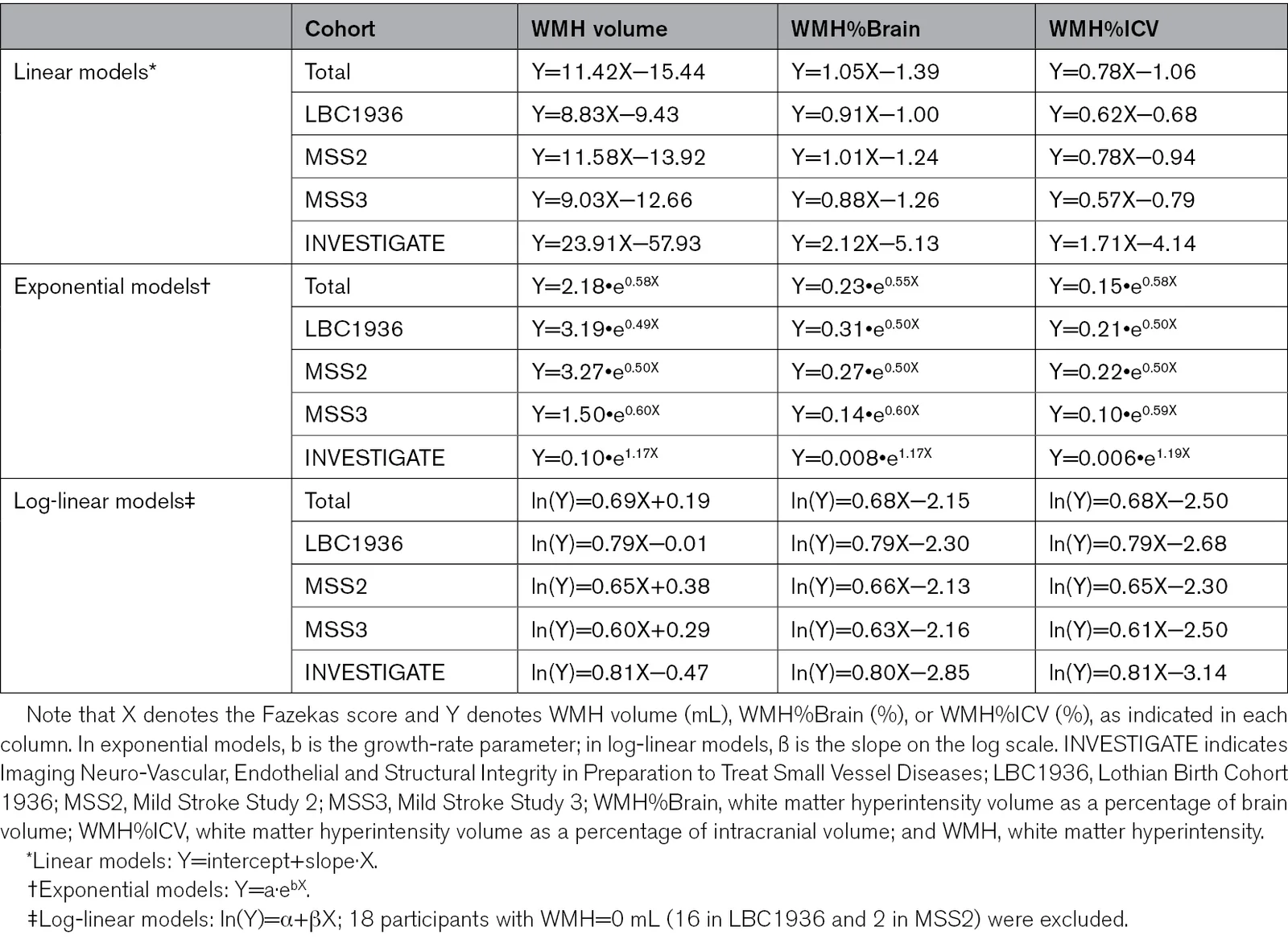
Linear and Exponential and Log-Linear Equations Relating the Fazekas Score to the WMH Volume, WMH%Brain, and WMH%ICV in the 4 Cohorts

Model fit indices (Table 3) generally favored the exponential formulation, with lower mean squared error values and support from Vuong test results, indicating that a nonlinear increase better captured the relationship between Fazekas score and WMH burden across cohorts.

**Table 3.**
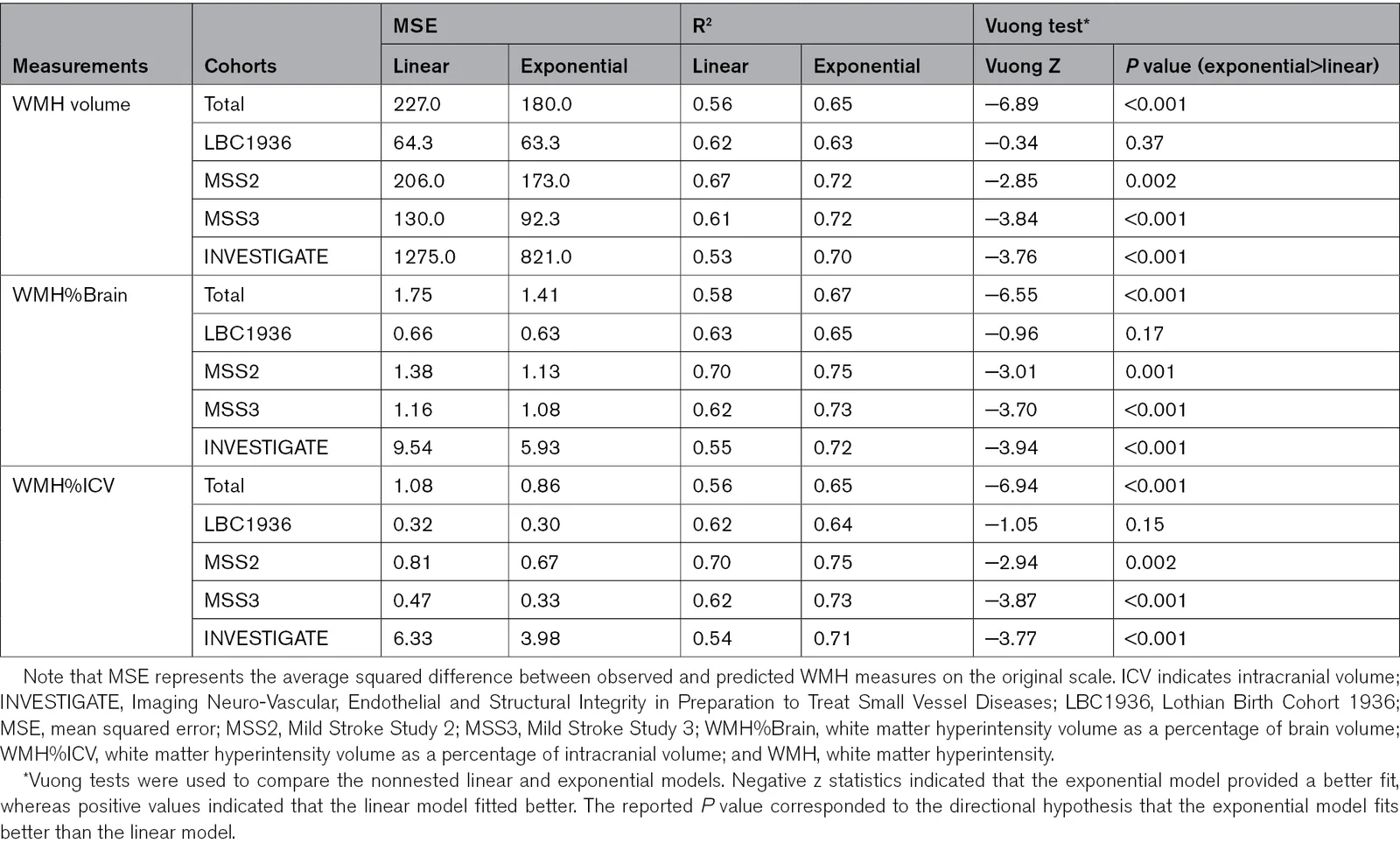
Model Fit Comparison of Linear and Exponential Equations Relating the WMH Volume and Normalized Ratios to the Fazekas Score to WMH Volume and Normalized WMH Ratios

### Log-Transformed WMH Measures (Sensitivity Analysis)

After exclusion of 18 participants with WMH volume=0 mL (16 in LBC1936 and 2 in MSS2), log-linear models again showed a strong monotonic association between Fazekas score and WMH burden (Table 2). Log-linear slopes (β) were generally steeper than the corresponding raw-scale exponential coefficients (b), most notably in the community-dwelling LBC1936 cohort (β=0.79 versus b=0.49), consistent with reduced influence of extreme high-volume observations on the log scale. Slope estimates were lowest in LBC1936, intermediate in the stroke cohorts (MSS2 and MSS3), and highest in INVESTIGATE, particularly for the raw-scale exponential model (b=1.17).

After back-transformation to the original WMH scale, the log-linear curves closely overlapped the NLS exponential curves through most of the Fazekas range (Figure S3). Model fit indices (Table S5) showed that the NLS models had consistently lower mean squared error values than the log-linear models on the original scale. However, because the log-linear and raw-scale NLS models were fitted on different scales and optimized different objective functions, these mean squared error comparisons should be interpreted cautiously.

### PVWMH and DWMH Contributions to WMH Volume, WMH%Brain, and WMH%ICV

Within a given total Fazekas score, different combinations of PVWMH and DWMH scores were not consistently associated with different WMH volume, WMH%Brain, or WMH%ICV. Although a small number of nominal pairwise differences were observed, the overall pattern was not consistent across WMH volume, WMH%Brain, and WMH%ICV (Table S6). These analyses should, nevertheless, be interpreted cautiously because several PVWMH/DWMH combinations were rare or absent.

## Discussion

This study bridged visual qualitative and quantitative volume assessments by linking the widely used, visually based Fazekas scores with computational measurements of WMH volume, and normalized ratios (WMH%Brain and WMH%ICV) using standardized WMH volume methods. Across the total sample and all 4 cohorts representing different CSVD presentations and severities (LBC1936, MSS2, MSS3, and INVESTIGATE), these metrics increase exponentially with rising Fazekas scores although the steepness of the progression differed by population.

### Visual Scale Versus Absolute Volume

The Fazekas scale separated WMH burden well, but overlap in WMH volume between adjacent grades was unavoidable. Consistent with earlier work,^8,9^ every 1-point increase in Fazekas was associated with a statistically significant jump in median WMH volume.

The largest overlaps were between grades 3 to 4 and 4 to 5; by contrast, a clear gap separated grades 5 and 6, suggesting a sharper biological or imaging threshold at the severe end. This ambiguity in the transition zones may have clinical implications, as individuals within these zones might represent a more heterogeneous group in terms of disease severity and risk, possibly indicating more difficulty in categorizing patients in moderate WMH categories; however, there were clearer distinctions in WMH volume between Fazekas scores of 2 and 3 (mild to moderate) and 4 and 5 (moderate to severe), representing clinically important categories.

### Population-Specific Growth Curves

By including multiple populations, a robust bidirectional conversion between WMH volume (and normalized ratios) and Fazekas scores was established using linear and exponential models. In most cohorts, the exponential model was preferable for most populations due to its higher explanatory power, highlighting the nonlinear increase in WMH burden, consistent with former studies.^27,28^ The differing steepness of the exponential curves across cohorts reflected differences in overall WMH severity, from the mildest to the most severe cohorts, and emphasized population-specific differences in WMH burden.

These patterns indicated that the same Fazekas grade does not correspond to the same WMH volume in all clinical settings.^29^ In cohorts with generally mild WMH, a Fazekas score of 3 tends to represent a smaller absolute lesion load than a Fazekas score of 3 in cohorts with more advanced small vessel disease. This perhaps reflected that visual raters apply the scale in a context-dependent way, effectively compressing or stretching their use of the 0 to 3 (or 0–6) range to match the range of severities they see in that population.

In addition, when rating scans visually, observers see WMH in the context of overall brain size and atrophy, whereas raw WMH volume did not account for this unless it was explicitly normalized by brain volume or ICV. The conversion equations we provided, therefore, offered a useful bridge between visual grades and quantitative volumes, particularly for large observational studies where manual rating was impractical and in clinical settings where automated segmentation was not yet widely available.

### Normalized Ratios

When interpreting our results, it was important to note that no gold standard currently existed for measuring WMH volume severity.^11^ Ratios normalized to ICV or brain volume might provide a more precise assessment of WMH burden than absolute WMH volume alone, while, in our study, normalization to brain volume or ICV did not materially change slope estimates within each cohort, indicating that the exponential relation is robust to head-size or brain atrophy adjustments. Moreover, previous studies had suggested that WMH worsening over time was associated with brain atrophy.^30,31^ Therefore, longitudinal investigations comparing changes in absolute WMH volume with changes in normalized WMH ratios were warranted to further assess the clinical value of WMH%Brain and WMH%ICV measures.

### PVWMH Versus DWMHs

We also examined the contributions of PVWMH and DWMH scores to the overall WMH volume burden. Within a given Fazekas grade, different PVWMH-DWMH combinations did not show clear or consistent differences in WMH volume or normalized ratios, suggesting that the global lesion burden, rather than regional predominance, was the main determinant of the visual score. The broadly similar PVWMH:DWMH proportions across grades provided no evidence of preferential expansion in one compartment as WMH burden increases. However, several PVWMH-DWMH categories were rare, so these analyses were underpowered and should be interpreted as exploratory rather than proof of equivalence.

### Log Transformation: Advantages and Caveats

Log-transforming WMH volumes (after excluding 18 zero-volume scans) produced steeper slopes (β) than the raw-scale NLS coefficients (b) in all the cohorts consistently because extreme values were downweighted on the log scale. The choice of scale therefore mattered: for etiological or prognostic models where homoscedastic residuals and multiplicative interpretation were desirable, log-WMH was advantageous and widely used.^32,33^ For mapping Fazekas grades to clinically intuitive milliliter ranges, raw-scale exponential fits remained preferable and avoided arbitrary constants for zero volumes. Researchers should recognize that log-models may underrepresent the heaviest lesions, and dropping zeros could bias low-burden strata.

### Strengths and Limitations

The strengths of this study included a large, prospective, population-based sample with a wide range of WMH volumes, which enhanced the generalizability of the findings for epidemiological research. By establishing a robust, bidirectional conversion between WMH volume (and normalized ratios) and Fazekas scores using both linear and exponential models, this study provided a valuable quantitative tool for large-scale observational studies and clinical practice. The studies were performed on one 1.5T (LBC1936 and MSS2) scanner or 3T (MSS3 and INVESTIGATE) magnetic resonance scanners, evaluated by the same team using consistent volumetric WMH measurement methods, thus minimizing between-individual and between-study sources of variance.

However, our study has several limitations. First, although the overall sample size was reasonably large, the equations were derived from a limited number of cohorts with cohort-specific inclusion criteria. These equations, therefore, summarize the average relationship between Fazekas grade and WMH burden within these exemplar data sets, rather than providing universally applicable conversion formulae, and they should not be used to infer precise WMH volumes for individual patients. External validation in larger and more diverse populations will be needed before wider use. Differences in demographics, risk factors, and imaging protocols might also limit how broadly these results apply. Second, some PVWMH/DWMH combination groups had few participants, which could reduce statistical power and increase the risk of type II errors. Third, exclusion of WMH=0 cases was required for the log-linear sensitivity analyses but may have affected cohort-specific slopes, particularly in LBC1936, where most zero-WMH cases occurred. Fourth, MRI acquisition and processing were not fully harmonized across cohorts (eg, 2-dimensional versus 3-dimensional FLAIR sequences, slice thickness, scanner, and segmentation differences), which might introduce systematic differences in WMH volume estimates between studies. Fifth, we used cohort-specific analyses because scanner generation and protocol differences may contribute to between-cohort differences in absolute WMH burden. Sixth, the limited ethnic diversity in our study population could restrict the applicability of the results to broader populations.

In conclusion, WMH volume and normalized ratios followed an exponential trajectory across Fazekas scores, but the progression patterns were population-dependent. The conversion equations provided enable bidirectional translation between visual Fazekas score and quantitative WMH volume, facilitating cross-study comparison, large-scale epidemiology, and clinical decision-making in CSVD.

## Article Information

### Acknowledgments

The Lothian Birth Cohort 1936 study acknowledges the financial support of NHS Research Scotland through the Edinburgh Clinical Research Facility. The authors acknowledge the Brain Research Imaging Center Radiography Team for magnetic resonance imaging acquisition at 1.5T and 3T. The authors also acknowledge the INVESTIGATE@SVDs Investigator Group in Munich and Maastricht.

### Disclosures

Dr Arteaga Reyes reports grants from Anne Rowling Clinic, the Mrs Gladys Row Fogo Charitable Trust, and the Mexican National Council of Humanities Sciences and Technology. Dr Clancy reports grants from the Chief Scientist Office and the Stroke Association. Dr Stringer reports employment by the University of Glasgow; employment by the College of Medicine and Veterinary Medicine, The University of Edinburgh; and grants from UK DRI Ltd, the Stroke Association, and the Horizon 2020 Framework Program. Dr Liu reports grants from Weill Neurohub. Dr Doubal reports grants from The Stroke Association-United Kingdom, the NHS Scotland Research, and the Agnes Parry Bequest. Dr Cox reports grants from the National Institute on Aging, Age UK, Wellcome Trust, the Biotechnology and Biological Sciences Research Council, and the Medical Research Council. Dr Kopczak reports grants from Deutsche Forschungsgemeinschaft and the European Commission to others. Dr Wardlaw reports grants from the Medical Research Council, Age UK, the Chief Scientist Office, the Dunhill Medical Trust, the Biotechnology and Biological Sciences Research Council, Alzheimer’s Society, Fondation Leducq, the Stroke Association, Wellcome Trust, British Heart Foundation, the Horizon 2020 Framework Program, the Mrs Gladys Row Fogo Charitable Trust, the Alzheimer’s Research UK, and the Weston Brain Institute and grants from Dunhill Trust, Research Councils UK, and the Scottish Funding Council to other services. The other authors report no conflicts.

### Supplemental Material

Tables S1–S6

Figures S1–S3

STROBE Checklist

## Supplementary Material



## References

[1] Wardlaw JM Smith EE Biessels GJ Cordonnier C Fazekas F Frayne R Lindley RI O’Brien JT Barkhof F Benavente OR; STandards for ReportIng Vascular changes on nEuroimaging (STRIVE v1). Neuroimaging standards for research into small vessel disease and its contribution to ageing and neurodegeneration. Lancet Neurol. 2013;12:822–838. doi: 10.1016/S1474-4422(13)70124-8 23867200 10.1016/S1474-4422(13)70124-8 PMC3714437

[2] Duering M Biessels GJ Brodtmann A Chen C Cordonnier C de Leeuw F-E Debette S Frayne R Jouvent E Rost NS. Neuroimaging standards for research into small vessel disease-advances since 2013. Lancet Neurol. 2023;22:602–618. doi: 10.1016/S1474-4422(23)00131-X 37236211 10.1016/S1474-4422(23)00131-X

[3] Smith EE Biessels GJ De Guio F de Leeuw FE Duchesne S Düring M Frayne R Ikram MA Jouvent E MacIntosh BJ. Harmonizing brain magnetic resonance imaging methods for vascular contributions to neurodegeneration. Alzheimers Dement (Amst). 2019;11:191–204. doi: 10.1016/j.dadm.2019.01.002 30859119 10.1016/j.dadm.2019.01.002 PMC6396326

[4] Fazekas F Chawluk JB Alavi A Hurtig HI Zimmerman RA. MR signal abnormalities at 1.5 T in Alzheimer’s dementia and normal aging. AJR Am J Roentgenol. 1987;149:351–356. doi: 10.2214/ajr.149.2.351 3496763 10.2214/ajr.149.2.351

[5] Caligiuri ME Perrotta P Augimeri A Rocca F Quattrone A Cherubini A. Automatic detection of white matter hyperintensities in healthy aging and pathology using magnetic resonance imaging: a review. Neuroinformatics. 2015;13:261–276. doi: 10.1007/s12021-015-9260-y 25649877 10.1007/s12021-015-9260-y PMC4468799

[6] Balakrishnan R Valdés Hernández MDC Farrall AJ. Automatic segmentation of white matter hyperintensities from brain magnetic resonance images in the era of deep learning and big data - a systematic review. Comput Med Imaging Graph. 2021;88:101867. doi: 10.1016/j.compmedimag.2021.101867 33508567 10.1016/j.compmedimag.2021.101867

[7] Valdés Hernández MDC Ferguson KJ Chappell FM Wardlaw JM. New multispectral MRI data fusion technique for white matter lesion segmentation: method and comparison with thresholding in FLAIR images. Eur Radiol. 2010;20:1684–1691. doi: 10.1007/s00330-010-1718-6 20157814 10.1007/s00330-010-1718-6 PMC2882045

[8] Andere A Jindal G Molino J Collins S Merck D Burton T Stretz C Yaghi S Sacchetti DC Jamal SE. Volumetric white matter hyperintensity ranges correspond to Fazekas scores on brain MRI. J Stroke Cerebrovasc Dis. 2022;31:106333. doi: 10.1016/j.jstrokecerebrovasdis.2022.106333 35158149 10.1016/j.jstrokecerebrovasdis.2022.106333

[9] Valdés Hernández MDC Morris Z Dickie DA Royle NA Muñoz Maniega S Aribisala BS Bastin ME Deary IJ Wardlaw JM. Close correlation between quantitative and qualitative assessments of white matter lesions. Neuroepidemiology. 2013;40:13–22. doi: 10.1159/000341859 23075702 10.1159/000341859

[10] Prins ND van Straaten ECW van Dijk EJ Simoni M van Schijndel RA Vrooman HA Koudstaal PJ Scheltens P Breteler MMB Barkhof F. Measuring progression of cerebral white matter lesions on MRI: visual rating and volumetrics. Neurology. 2004;62:1533–1539. doi: 10.1212/01.wnl.0000123264.40498.b6 15136677 10.1212/01.wnl.0000123264.40498.b6

[11] Gouw AA van der Flier WM van Straaten ECW Pantoni L Bastos-Leite AJ Inzitari D Erkinjuntti T Wahlund LO Ryberg C Schmidt R; LADIS Study Group. Reliability and sensitivity of visual scales versus volumetry for evaluating white matter hyperintensity progression. Cerebrovasc Dis. 2008;25:247–253. doi: 10.1159/000113863 18216467 10.1159/000113863

[12] Enzinger C Fazekas F Ropele S Schmidt R. Progression of cerebral white matter lesions — clinical and radiological considerations. J Neurol Sci. 2007;257:5–10. doi: 10.1016/j.jns.2007.01.018 17321549 10.1016/j.jns.2007.01.018

[13] Gouw AA van der Flier WM van Straaten ECW Barkhof F Ferro JM Baezner H Pantoni L Inzitari D Erkinjuntti T Wahlund LO; LADIS Study Group. Simple versus complex assessment of white matter hyperintensities in relation to physical performance and cognition: the LADIS study. J Neurol. 2006;253:1189–1196. doi: 10.1007/s00415-006-0193-5 16998647 10.1007/s00415-006-0193-5

[14] Maniega SM Valdés Hernández MDC Clayden JD Royle NA Murray C Morris Z Aribisala BS Gow AJ Starr JM Bastin ME. White matter hyperintensities and normal-appearing white matter integrity in the aging brain. Neurobiol Aging. 2015;36:909–918. doi: 10.1016/j.neurobiolaging.2014.07.048 25457555 10.1016/j.neurobiolaging.2014.07.048 PMC4321830

[15] Kilinc D Vernooij MW Bron EE Biessels GJ Vinke EJ. Normative population-derived data for MRI manifestations of cerebral small vessel disease. Stroke. 2024;55:2863–2871. doi: 10.1161/STROKEAHA.124.046731 39585934 10.1161/STROKEAHA.124.046731

[16] Deary IJ Gow AJ Taylor MD Corley J Brett C Wilson V Campbell H Whalley LJ Visscher PM Porteous DJ. The Lothian Birth Cohort 1936: a study to examine influences on cognitive ageing from age 11 to age 70 and beyond. BMC Geriatr. 2007;7:28. doi: 10.1186/1471-2318-7-28 18053258 10.1186/1471-2318-7-28 PMC2222601

[17] Valdés Hernández MDC Armitage PA Thrippleton MJ Chappell F Sandeman E Muñoz Maniega S Shuler K Wardlaw JM. Rationale, design and methodology of the image analysis protocol for studies of patients with cerebral small vessel disease and mild stroke. Brain Behav. 2015;5:e00415. doi: 10.1002/brb3.415 26807340 10.1002/brb3.415 PMC4714639

[18] Clancy U Garcia DJ Stringer MS Thrippleton MJ Valdés Hernández MDC Wiseman S Hamilton OK Chappell FM Brown R Blair GW. Rationale and design of a longitudinal study of cerebral small vessel diseases, clinical and imaging outcomes in patients presenting with mild ischaemic stroke: Mild Stroke Study 3. Eur Stroke J. 2021;6:81–88. doi: 10.1177/2396987320929617 33817338 10.1177/2396987320929617 PMC7995323

[19] Blair GW Stringer MS Thrippleton MJ Chappell FM Shuler K Hamilton I Garcia DJ Doubal FN Kopczak A Duering M. Imaging neurovascular, endothelial and structural integrity in preparation to treat small vessel diseases. The INVESTIGATE-SVDs study protocol. Part of the SVDs@Target project. Cereb Circ Cogn Behav. 2021;2:100020. doi: 10.1016/j.cccb.2021.100020 36324725 10.1016/j.cccb.2021.100020 PMC9616332

[20] Luciano M Marioni RE Valdés Hernández MDC Muñoz Maniega S Hamilton IF Royle NA Chauhan G Bis JC Debette S Decarli CS; Generation Scotland. Structural brain MRI trait polygenic score prediction of cognitive abilities. Twin Res Hum Genet. 2015;18:738–745. doi: 10.1017/thg.2015.71 26427786 10.1017/thg.2015.71 PMC4747328

[21] Makin SDJ Doubal FN Dennis MS Wardlaw JM. Clinically confirmed stroke with negative diffusion-weighted imaging magnetic resonance imaging: longitudinal study of clinical outcomes, stroke recurrence, and systematic review. Stroke. 2015;46:3142–3148. doi: 10.1161/STROKEAHA.115.010665 26419965 10.1161/STROKEAHA.115.010665 PMC4617292

[22] Jenkinson M Bannister P Brady M Smith S. Improved optimization for the robust and accurate linear registration and motion correction of brain images. Neuroimage. 2002;17:825–841. doi: 10.1016/s1053-8119(02)91132-8 12377157 10.1016/s1053-8119(02)91132-8

[23] Zhang Y Brady M Smith S. Segmentation of brain MR images through a hidden Markov random field model and the expectation-maximization algorithm. IEEE Trans Med Imaging. 2001;20:45–57. doi: 10.1109/42.906424 11293691 10.1109/42.906424

[24] Jenkinson M Péchaud M Smith S. BET2: MR-Based Estimation of Brain, Skull and Scalp Surfaces. In: Eleventh Annual Meeting of the Organization for Human Brain Mapping. 2005.

[25] Chen L Tong T Ho CP Patel R Cohen D Dawson AC Halse O Geraghty O Rinne PEM White CJ. Identification of Cerebral Small Vessel Disease Using Multiple Instance Learning. In: Machine Learning in Medical Imaging. 2015:523–530.

[26] Vuong QH. Likelihood ratio tests for model selection and non-nested hypotheses. Econometrica. 1989;57:307–333. doi: 10.2307/1912557

[27] van Straaten ECW Fazekas F Rostrup E Scheltens P Schmidt R Pantoni L Inzitari D Waldemar G Erkinjuntti T Mäntylä R; LADIS Group. Impact of white matter hyperintensities scoring method on correlations with clinical data: the LADIS study. Stroke. 2006;37:836–840. doi: 10.1161/01.STR.0000202585.26325.74 16439704 10.1161/01.STR.0000202585.26325.74

[28] van Leijsen EMC van Uden IWM Ghafoorian M Bergkamp MI Lohner V Kooijmans ECM van der Holst HM Tuladhar AM Norris DG van Dijk EJ. Nonlinear temporal dynamics of cerebral small vessel disease: the RUN DMC study. Neurology. 2017;89:1569–1577. doi: 10.1212/WNL.0000000000004490 28878046 10.1212/WNL.0000000000004490 PMC5634663

[29] Wardlaw JM Ferguson KJ Graham C. White matter hyperintensities and rating scales-observer reliability varies with lesion load. J Neurol. 2004;251:584–590. doi: 10.1007/s00415-004-0371-x 15164192 10.1007/s00415-004-0371-x

[30] Kloppenborg RP Nederkoorn PJ Grool AM Vincken KL Mali WPTM Vermeulen M van der Graaf Y Geerlings MI; SMART Study Group. Cerebral small-vessel disease and progression of brain atrophy: the SMART-MR study. Neurology. 2012;79:2029–2036. doi: 10.1212/WNL.0b013e3182749f02 23115210 10.1212/WNL.0b013e3182749f02

[31] Appelman APA Exalto LG van der Graaf Y Biessels GJ Mali WPTM Geerlings MI. White matter lesions and brain atrophy: more than shared risk factors? A systematic review. Cerebrovasc Dis. 2009;28:227–242. doi: 10.1159/000226774 19571536 10.1159/000226774

[32] Sheibani N Wong K-H Turan TN Yeatts SD Gottesman RF Prabhakaran S Rost NS de Havenon A. White matter hyperintensity and cardiovascular disease outcomes in the SPRINT MIND trial. J Stroke Cerebrovasc Dis. 2021;30:105764. doi: 10.1016/j.jstrokecerebrovasdis.2021.105764 33823461 10.1016/j.jstrokecerebrovasdis.2021.105764 PMC8107132

[33] Wang J Ackley S Woodworth DC Sajjadi SA Decarli CS Fletcher EF Glymour MM Jiang L Kawas C Corrada MM. Associations of amyloid Burden, white matter hyperintensities, and hippocampal volume with cognitive trajectories in the 90+ study. Neurology. 2024;103:e209665. doi: 10.1212/WNL.0000000000209665 39008782 10.1212/WNL.0000000000209665 PMC11249511

