# Supplementary material for: Quantitative Relationship Between White Matter Hyperintensity Volume and Fazekas Score on Brain MRI
Source: Stroke. 2026 Jul 15;57(9):2801–12. doi: 10.1161/STROKEAHA.125.055101 (PMC13506189; doi:10.1161/STROKEAHA.125.055101)
Supplement: Supplementary file 1 [file str-57-2801-s001.pdf]

SUPPLEMENTAL MATERIAL

Table S1. Co-enrolled participants ID

MSS3 ID	INVESTIGATE ID
011	716
037	720
040	721
043	722
045	723
046	724
049	725

Note: MSS3–INVESTIGATE co-enrolments (n=7). Cohorts were analyzed separately; pooled unique-individual counts deduct these pairs.

Table S2. The WMH volume, WMH%Brain, and WMH%ICV for each Fazekas score in four cohorts: mean±SD and median (IQR)

	Fazekas		Total N=1227	LBC1936 N=658	MSS2 N=264	MSS3 N=228	INVESTIGATE N=77	P (Anova, 4 groups)	P (KW, 4 groups)
**WMH vol, ml**	0	Median (IQR)	0.54 (0.01–1.27)	0 (0–0.06)	0.73 (0.46–1.87)	1.41 (1.22–1.90)	NA	0.010	0.002
		Mean±SD	0.89±1.06	0.11±0.24	1.26±1.25	1.57±0.76	NA		
	1	Median (IQR)	2.30 (0.92–4.66)	1.78 (0.60–4.60)	2.76 (1.37–5.21)	3.13 (2.21–5.13)	3.71 (3.63–3.78)	0.113	0.067
		Mean±SD	3.06±2.81	2.68±2.56	3.71±3.49	4.12±2.91	3.71±0.20		
	2	Median (IQR)	5.05 (2.97–8.39)	5.49 (3.16–9.07)	5.33 (3.15–11.12)	4.16 (2.29–5.99)	4.03 (1.89–5.00)	<0.001	<0.001
		Mean±SD	6.51±5.14	6.80±5.17	7.51±6.13	4.93±3.61	3.85±2.37		
	3	Median (IQR)	12.68 (8.28–18.99)	15.04 (10.58–21.46)	12.66 (9.65–17.98)	8.78 (6.56–11.33)	7.53 (5.35–8.82)	<0.001	<0.001
		Mean±SD	14.34±7.75	16.15±7.95	14.74±7.55	9.40±4.03	7.06±2.20		
	4	Median (IQR)	21.21 (15.07–31.62)	22.53 (17.76–34.52)	28.84 (19.64–35.85)	16.84 (13.71–21.97)	12.38 (11.12–13.65)	<0.001	<0.001
		Mean±SD	23.69±11.50	25.38±11.35	28.13±12.68	18.25±7.17	13.61±5.75		
	5	Median (IQR)	29.78 (24.77–39.42)	31.76 (28.31–40.76)	37.40 (27.87–42.62)	26.22 (21.59–29.57)	28.25 (18.65–35.07)	0.004	0.002
		Mean±SD	33.18±13.64	36.66±16.27	37.88±14.02	26.82±7.91	28.96±11.02		
	6	Median (IQR)	59.83 (43.51–89.67)	45.60 (41.99–59.74)	59.83 (43.60–81.04)	46.66 (36.92–70.60)	95.99 (65.99–150.07)	<0.001	0.002
		Mean±SD	70.46±38.24	53.89±19.40	64.17±25.86	55.05±26.69	105.47±51.38		
**WMH %Brain**	0	Median (IQR)	0.05 (0.001–0.11)	0 (0–0.01)	0.06 (0.04–0.14)	0.12 (0.11–0.16)	NA	0.013	0.003
		Mean±SD	0.07±0.09	0.01±0.03	0.10±0.10	0.13±0.05	NA		
	1	Median (IQR)	0.21 (0.09–0.46)	0.18 (0.06–0.47)	0.18 (0.11–0.42)	0.27 (0.17–0.42)	0.34 (0.32–0.36)	0.616	0.284
		Mean±SD	0.29±0.27	0.28±0.26	0.31±0.30	0.36±0.27	0.34±0.05		
	2	Median (IQR)	0.49 (0.28–0.83)	0.56 (0.33–0.92)	0.41 (0.27–0.96)	0.36 (0.20–0.55)	0.38 (0.17–0.45)	<0.001	<0.001
		Mean±SD	0.62±0.48	0.69±0.50	0.63±0.51	0.45±0.34	0.35±0.22		
	3	Median (IQR)	1.17 (0.78–1.86)	1.50 (1.03–2.13)	1.05 (0.80–1.45)	0.83 (0.64–1.16)	0.70 (0.49–0.82)	<0.001	<0.001
		Mean±SD	1.38±0.76	1.61±0.79	1.21±0.62	0.90±0.38	0.65±0.20		
	4	Median (IQR)	2.12 (1.42–2.88)	2.30 (1.81–3.60)	2.47 (1.62–3.08)	1.68 (1.27–2.12)	1.17 (1.11–1.35)	<0.001	<0.001
		Mean±SD	2.28±1.11	2.60±1.16	2.40±1.03	1.76±0.80	1.25±0.38		
	5	Median (IQR)	2.92 (2.32–3.69)	3.23 (2.58–4.14)	3.15 (2.61–3.85)	2.79 (1.98–3.36)	2.67 (1.63–2.97)	0.003	0.009
		Mean±SD	3.16±1.35	3.78±1.83	3.36±1.18	2.65±0.77	2.53±0.89		

	6	Median (IQR)	5.75 (4.16–7.68)	5.25 (4.79–6.06)	4.93 (3.75–6.90)	4.35 (3.34–7.22)	8.49 (6.15–13.66)	<0.001	<0.001
		Mean±SD	6.40±3.24	5.70±1.60	5.54±2.06	5.27±2.50	9.39±4.37		
**WMH %ICV**	0	Median (IQR)	0.04 (0.001–0.08)	0 (0–0.005)	0.05 (0.03–0.12)	0.08 (0.08–0.12)	NA	0.012	0.003
		Mean±SD	0.06±0.07	0.01±0.02	0.09±0.08	0.09±0.04	NA		
	1	Median (IQR)	0.15 (0.06–0.31)	0.12 (0.04–0.31)	0.16 (0.09–0.33)	0.19 (0.13–0.29)	0.25 (0.24–0.26)	0.306	0.175
		Mean±SD	0.21±0.19	0.19±0.18	0.25±0.24	0.26±0.19	0.25±0.03		
	2	Median (IQR)	0.34 (0.20–0.57)	0.39 (0.23–0.63)	0.34 (0.22–0.69)	0.25 (0.14–0.37)	0.29 (0.14–0.35)	<0.001	<0.001
		Mean±SD	0.44±0.34	0.47±0.34	0.50±0.40	0.30±0.21	0.27±0.17		
	3	Median (IQR)	0.84 (0.56–1.30)	1.01 (0.69–1.46)	0.85 (0.66–1.19)	0.57 (0.44–0.77)	0.50 (0.39–0.61)	<0.001	<0.001
		Mean±SD	0.96±0.52	1.09±0.54	0.98±0.47	0.59±0.23	0.49±0.14		
	4	Median (IQR)	1.41 (1.00–2.10)	1.60 (1.22–2.35)	1.85 (1.26–2.29)	1.06 (0.87–1.35)	0.90 (0.87–1.00)	<0.001	<0.001
		Mean±SD	1.60±0.77	1.76±0.78	1.87±0.80	1.13±0.41	1.00±0.34		
	5	Median (IQR)	2.13 (1.64–2.71)	2.27 (1.81–2.86)	2.62 (2.15–3.05)	1.78 (1.28–2.05)	1.94 (1.29–2.55)	0.001	<0.001
		Mean±SD	2.27±0.97	2.58±1.28	2.60±0.85	1.74±0.48	2.00±0.75		
	6	Median (IQR)	4.01 (3.04–5.98)	3.39 (3.29–4.14)	3.89 (3.09–5.27)	2.72 (2.36–4.77)	6.73 (5.16–10.53)	<0.001	<0.001
		Mean±SD	4.80±2.67	3.93±1.26	4.28±1.60	3.40±1.62	7.53±3.58		

Note: WMH: white matter hyperintensity. ICV: intracranial volume. SD: standard deviation. IQR: interquartile range. LBC: the Lothian Birth Cohort 1936. MSS2: Mild Stroke Study 2. MSS3: Mild Stroke Study 3. INVESTIGATE: Imaging Neuro-Vascular, Endothelial & STructural InteGrity in prepAration to TrEat Small Vessel Diseases.

Table S3. The value and the log transformed value of WMH volume, WMH%Brain, and WMH%ICV for each Fazekas score in the three groups (Community-dwelling, Stroke and CADASIL): median (IQR)

	Fazekas	Community-dwelling<br>N=658	Stroke<br>N=537	CADASIL<br>N=32
**WMH vol, ml**	0	0 (0–0.06)	1.07 (0.54–2.02)	/
	1	1.78 (0.60–4.60)	2.86 (1.94–5.16)	/
	2	5.49 (3.16–9.07)	4.54 (2.70–7.48)	/
	3	15.0 (10.60–21.5)	9.76 (7.37–12.8)	3.75 (3.75–3.75)
	4	22.50 (17.80–34.5)	19.0 (13.1–29.3)	19.0 (16.1–24.1)
	5	31.80 (28.30–40.8)	28.8 (23.5–39.2)	34.9 (28.3–41.9)
	6	45.6 (42.0–59.7)	52.3 (40.1–80.2)	96.0 (67.9–155.0)
**WMH%Brain**	0	0 (0–0.007)	0.09 (0.05–0.16)	/
	1	0.18 (0.06–0.47)	0.26 (0.16–0.42)	/
	2	0.56 (0.33–0.92)	0.39 (0.23–0.66)	/
	3	1.50 (1.03–2.13)	0.88 (0.68–1.16)	0.32 (0.32–0.32)
	4	2.30 (1.81–3.60)	1.76 (1.27–2.51)	1.72 (1.45–1.94)
	5	3.23 (2.58–4.14)	2.82 (2.04–3.43)	3.01 (2.54–3.68)
	6	5.25 (4.79–6.06)	4.78 (3.58–7.06)	8.49 (6.38–14.20)
**WMH%ICV**	0	0 (0–0.005)	0.08 (0.04–0.13)	/
	1	0.12 (0.04–0.31)	0.19 (0.11–0.29)	/
	2	0.39 (0.23–0.64)	0.29 (0.18–0.51)	/
	3	1.01 (0.69–1.46)	0.65 (0.48–0.87)	0.26 (0.26–0.26)
	4	1.60 (1.22–2.35)	1.23 (0.91–1.88)	1.46 (1.22–1.64)
	5	2.27 (1.81–2.86)	2.01 (1.43–2.54)	2.57 (2.10–3.00)
	6	3.39 (3.29–4.14)	3.51 (2.65–5.08)	6.91 (5.36–11.10)
**ln (WMH vol)**	0	-2.30 (-3.22–-1.42)	0.13 (-0.55–0.73)	/
	1	0.74 (-0.05–1.55)	1.06 (0.67–1.65)	/
	2	1.70 (1.16–2.21)	1.51 (1.00–2.01)	/
	3	2.71 (2.36–3.07)	2.28 (2.00–2.55)	1.32 (1.32–1.32)
	4	3.11 (2.88–3.54)	2.94 (2.57–3.38)	2.94 (2.76–3.16)
	5	3.46 (3.34–3.71)	3.36 (3.16–3.67)	3.55 (3.34–3.73)
	6	3.82 (3.74–4.09)	3.96 (3.69–4.38)	4.56 (4.22–5.05)
**ln (WMH%Brain)**	0	-4.52 (-5.45–-3.68)	-2.30 (-3.02–-1.84)	/
	1	-1.48 (-2.40–-0.72)	-1.31 (-1.85– -0.87)	/
	2	-0.57 (-1.11–-0.08)	-0.95 (-1.49–-0.41)	/
	3	0.41 (0.03–0.75)	-0.13 (-0.39–0.15)	-1.14 (-1.14–-1.14)
	4	0.83 (0.59–1.28)	0.57 (0.24–0.92)	0.55 (0.35–0.65)
	5	1.17 (0.95–1.42)	1.04 (0.72–1.23)	1.10 (0.93–1.30)
	6	1.66 (1.57–1.80)	1.56 (1.27–1.95)	2.14 (1.85–2.65)
**ln (WMH%ICV)**	0	-4.86 (-5.70–-4.05)	-2.54 (-3.19–-2.05)	/
	1	-1.89 (-2.79–-1.09)	-1.60 (-2.18–-1.23)	/
	2	-0.93 (-1.48–-0.45)	-1.22 (-1.74–-0.68)	/
	3	0.01 (-0.37–0.38)	-0.43 (-0.74–-0.14)	-1.35 (-1.35–-1.35)

4	0.47 (0.20–0.86)	0.20 (-0.10–0.63)	0.38 (0.18–0.49)
5	0.82 (0.60–1.05)	0.70 (0.36–0.93)	0.94 (0.73–1.10)
6	1.22 (1.19–1.42)	1.26 (0.98–1.63)	1.93 (1.68–2.41)

Note: To convert to the log-10 transformed value, multiply the ln-transformed value by 0.434.

WMH: white matter hyperintensity. ICV: intracranial volume.

Table S4. Differences in WMH volume between adjacent Fazekas scores in the total population and each cohort.

**Comparison of WMH volume**	**Total population**		**LBC1936**		**MSS2**		**MSS3**		**INVESTIGATE**	
	Difference (95% CI)	P value	Difference (95% CI)	P value	Difference (95% CI)	P value	Difference (95% CI)	P value	Difference (95% CI)	P value
**Fazekas 0 vs 1**	-1.51 (-2.48–-0.76)	<0.001	-1.65 (-3.97–-0.66)	<0.001	-1.60 (-2.98–-0.00)	0.05	-1.60 (-5.66–-0.31)	0.02	NA	NA
**Fazekas 1 vs 2**	-2.65 (-3.24–-2.06)	<0.001	-3.38 (-4.15–-2.63)	<0.001	-2.65 (-4.41–-1.15)	<0.001	-0.52 (-1.87–0.52)	0.37	-0.33 (-5.75–2.93)	0.94
**Fazekas 2 vs 3**	-6.97 (-7.92–-6.08)	<0.001	-8.93 (-10.27–-7.63)	<0.001	-6.78 (-8.87–-4.79)	<0.001	-4.50 (-5.68–-3.30)	<0.001	-3.60 (-5.06–-1.25)	0.003
**Fazekas 3 vs 4**	-8.34 (-10.37–-6.37)	<0.001	-8.64 (-11.60–-5.65)	<0.001	-13.73 (-19.19–-8.66)	<0.001	-7.87 (-10.61–-5.50)	<0.001	-5.26 (-8.22–-3.01)	<0.001
**Fazekas 4 vs 5**	-9.06 (-11.84–-6.18)	<0.001	-9.83 (-15.02–-4.33)	<0.001	-9.31 (-15.23–-2.03)	0.01	-8.80 (-12.68–-5.07)	<0.001	-14.88 (-22.98–-6.18)	<0.001
**Fazekas 5 vs 6**	-28.14 (-36.39–-21.93)	<0.001	-14.77 (-24.84–-5.87)	0.001	-23.05 (-33.82–-13.01)	<0.001	-22.02 (-33.91–-12.88)	<0.001	-65.43 (-103.93–-40.52)	<0.001

Note: WMH: white matter hyperintensity. CI: confidence intervals. LBC1936: the Lothian Birth Cohort 1936. MSS2: Mild Stroke Study 2. MSS3: Mild Stroke Study 3. INVESTIGATE: Imaging Neuro-Vascular, Endothelial & STructural InteGrity in prepAration to TrEat Small Vessel Diseases.

Table S5. Model fit indices (MSE and R^2) of log-linear and exponential (NLS) models in the total population and four cohorts

**Measurements**	**Groups**	**MSE (prediction error)**		***R^2***	***Pseudo-R^2***
		**Log-linear**	**NLS**	***Log-linear***	***NLS***
**WMH volume**	Total	187.25	180.30	0.61	0.65
	LBC1936	144.62	63.27	0.49	0.62
	MSS2	191.69	173.25	0.70	0.72
	MSS3	96.03	92.26	0.73	0.72
	INVESTIGATE	1046.84	820.63	0.85	0.70
**WMH%Brain**	Total	1.51	1.41	0.61	0.66
	LBC1936	1.42	0.63	0.49	0.64
	MSS2	1.30	1.13	0.66	0.75
	MSS3	0.85	0.83	0.63	0.73
	INVESTIGATE	7.61	5.93	0.80	0.72
**WMH%ICV**	Total	0.89	0.86	0.61	0.65
	LBC1936	0.65	0.30	0.49	0.64
	MSS2	0.76	0.67	0.71	0.75
	MSS3	0.34	0.33	0.75	0.74
	INVESTIGATE	5.09	3.98	0.86	0.71

Note: Mean squared error (MSE) represents the average squared difference between observed and predicted WMH measures on the original scale. Pseudo-R^2 (for NLS) and R^2 (for log-linear) indicate the proportion of variance explained. Lower MSE indicates lower prediction error on the original scale; R^2 and pseudo-R^2 are reported descriptively and should not be directly compared across model types. Patients with 0mL WMH volume (n=18) were excluded.

Table S6. PVWMH and DWMH Contributions to WMH volume, WMH%Brain, and WMH%ICV in the total population

A. Differences in WMH volume, WMH/Brain, and WMH%ICV between groups

Fazekas	PVWMH	DWMH	No. of participants	WMH, ml mean±SD	P	WMH%ICV mean±SD	P	WMH%Brain mean±SD	P
0	0	0	24	0.89±1.06	/	0.06±0.07	/	0.07±0.09	/
1	0	1	26	2.25±2.65	0.05	0.15±0.17	0.06	0.21±0.25	0.04
	1	0	118	3.24±2.82		0.22±0.19		0.31±0.27	
2	1	1	490	6.45±5.07	0.08	0.44±0.33	0.08	0.62±0.48	0.06
	2	0	4	13.0±9.93		0.81±0.58		1.24±0.92	
3	1	2	59	14.1±7.30	0.24	0.97±0.50	0.22	1.37±0.72	0.23
	2	1	162	14.3±7.76		0.95±0.52		1.37±0.76	
	3	0	1	36.2		2.32		3.45	
4	1	3	2	25.3±11.8	0.07	1.55±0.77	0.09	1.99±0.74	0.06
	2	2	131	23.1±11.5		1.56±0.76		2.22±1.09	
	3	1	9	31.3±10.6		2.13±0.75		3.11±1.09	
5	2	3	18	35.9±18.1	0.53	2.56±1.38	0.34	3.46±1.94	0.76
	3	2	80	32.6±12.5		2.20±0.85		3.10±1.18	
6	3	3	103	70.5±38.2	/	4.80±2.67	/	6.40±3.24	/

B. Pairwise comparison of WMH volume, WMH%Brain, and WMH%ICV between groups

Fazekas	Pairwise comparison (PVWMH+ DWMH)	P value WMH volume	P value WMH%ICV	P value WMH%Brain
0	/	/	/	/
1	(0+1) vs (1+0)	0.05	0.06	0.04
2	(1+1) vs (2+0)	0.08	0.08	0.06
3	(1+2) vs (2+1)	1.00	1.00	1.00
	(1+2) vs (3+0)	0.28	0.28	0.28
	(2+1) vs (3+0)	0.29	0.29	0.29
4	(2+2) vs (3+1)	0.06	0.08	0.06
	(2+2) vs (1+3)	1.00	1.00	1.00
	(1+3) vs (3+1)	1.00	1.00	0.98
5	(2+3) vs (3+2)	0.54	0.34	0.77
6	/	/	/	/

SD: standard deviation. WMH: white matter hyperintensity. PVWMH: periventricular white matter hyperintensity. DWMH: deep white matter hyperintensity. ICV: intracranial volume. LBC1936: the Lothian Birth Cohort 1936. MSS2: Mild Stroke Study 2. MSS3: Mild Stroke Study 3. INVESTIGATE: Imaging Neuro-Vascular, Endothelial & STructural InteGrity in prepAration to TrEat Small Vessel Diseases.

Figure S1. Distribution of Fazekas scores across cohorts.

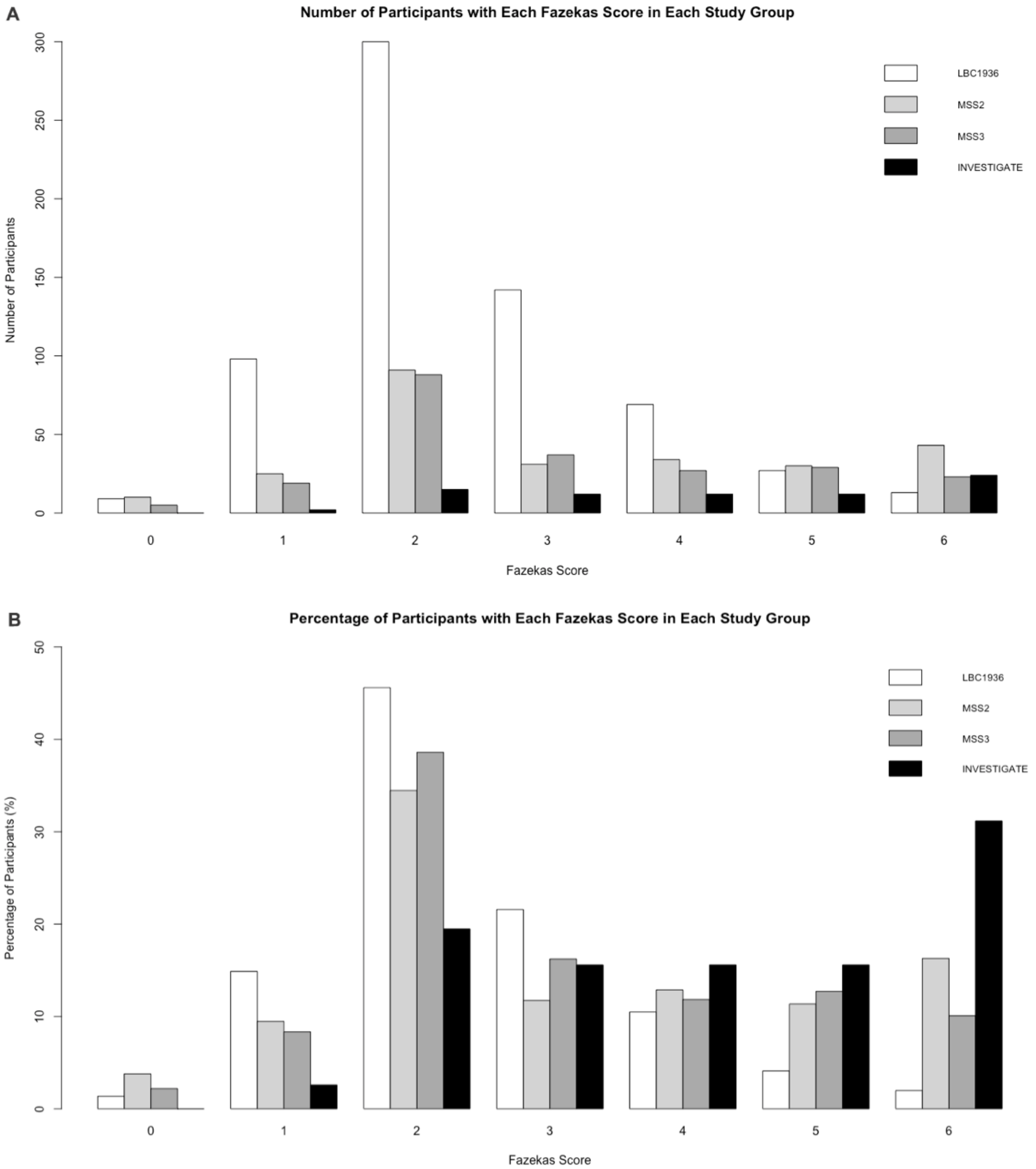

Number (Figure S1A) and percentage (Figure S1B) of participants with each total Fazekas score in LBC1936, MSS2, MSS3, and INVESTIGATE.

LBC1936: the Lothian Birth Cohort 1936. MSS2: Mild Stroke Study 2. MSS3: Mild Stroke Study 3. INVESTIGATE: Imaging Neuro-Vascular, Endothelial & STructural InteGrity in prepAration to TrEat Small Vessel Diseases.

Figure S2. The transition zones of the WMH volume, WMH%Brain, and WMH%ICV between adjacent Fazekas scores in the total population

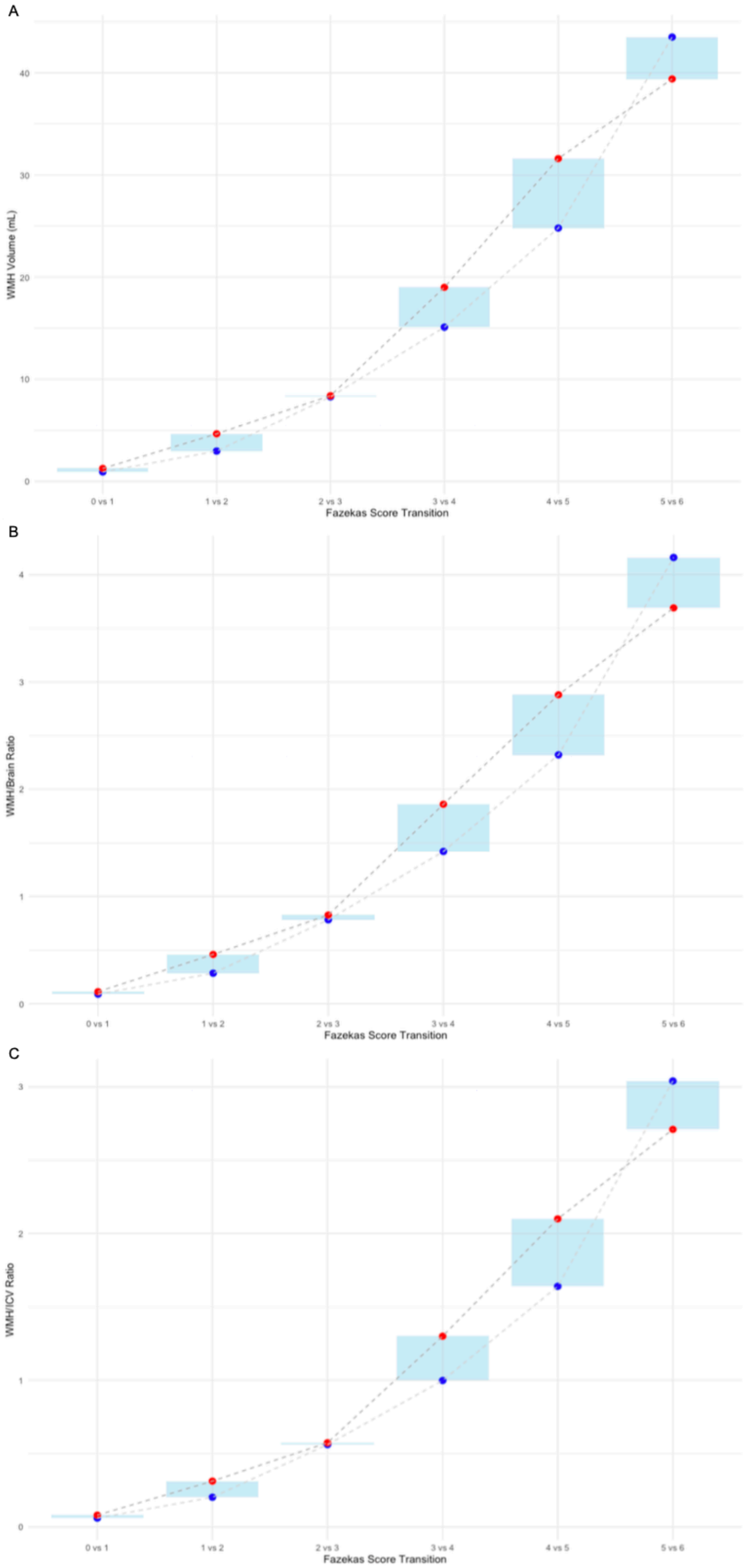

Panels show (A) WMH volume, (B) WMH%Brain, and (C) WMH%ICV. Transition zones are defined as the overlap of interquartile ranges between adjacent Fazekas scores. Shaded light-blue rectangles indicate transition zones; blue dots indicate transition start values (Q1), red dots indicate transition end values (Q3), and dashed grey lines connect start and end values.

Figure S3. Overlay of NLS exponential fits and back-transformed log-linear fits for WMH measures in the total population and each cohort.

WMH vol, ml	WMH%Brain	WMH%ICV

Total population

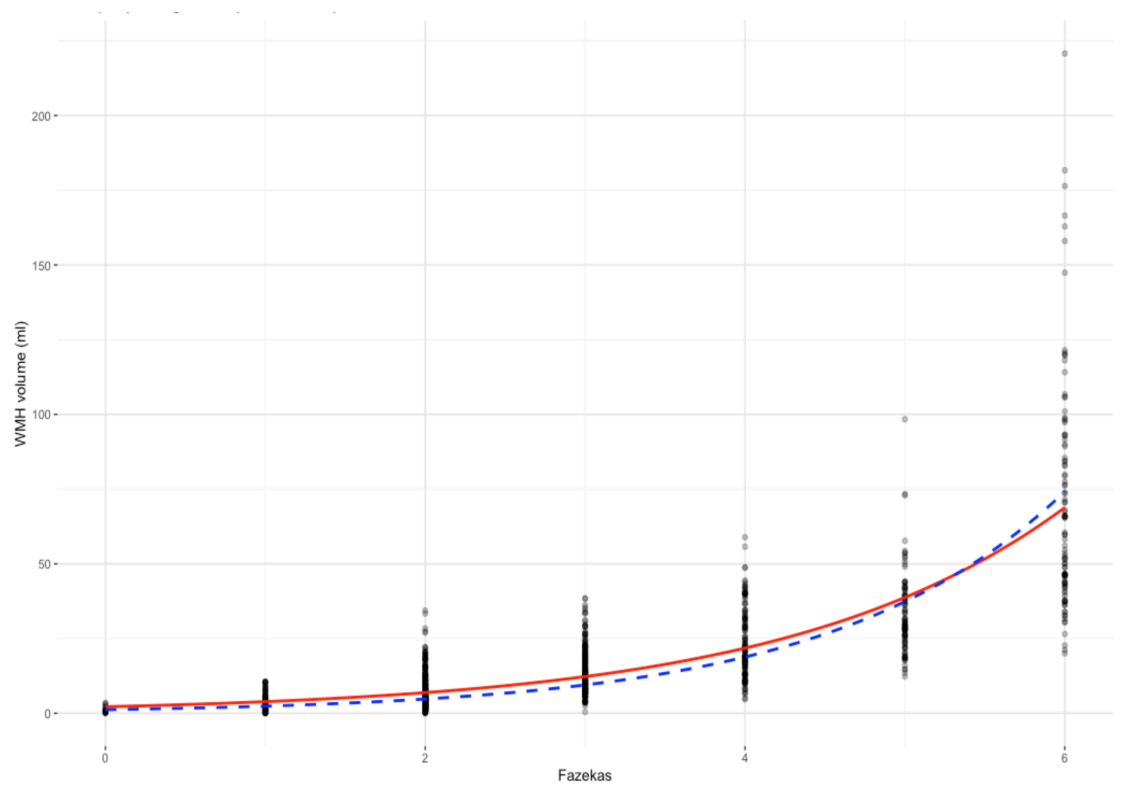

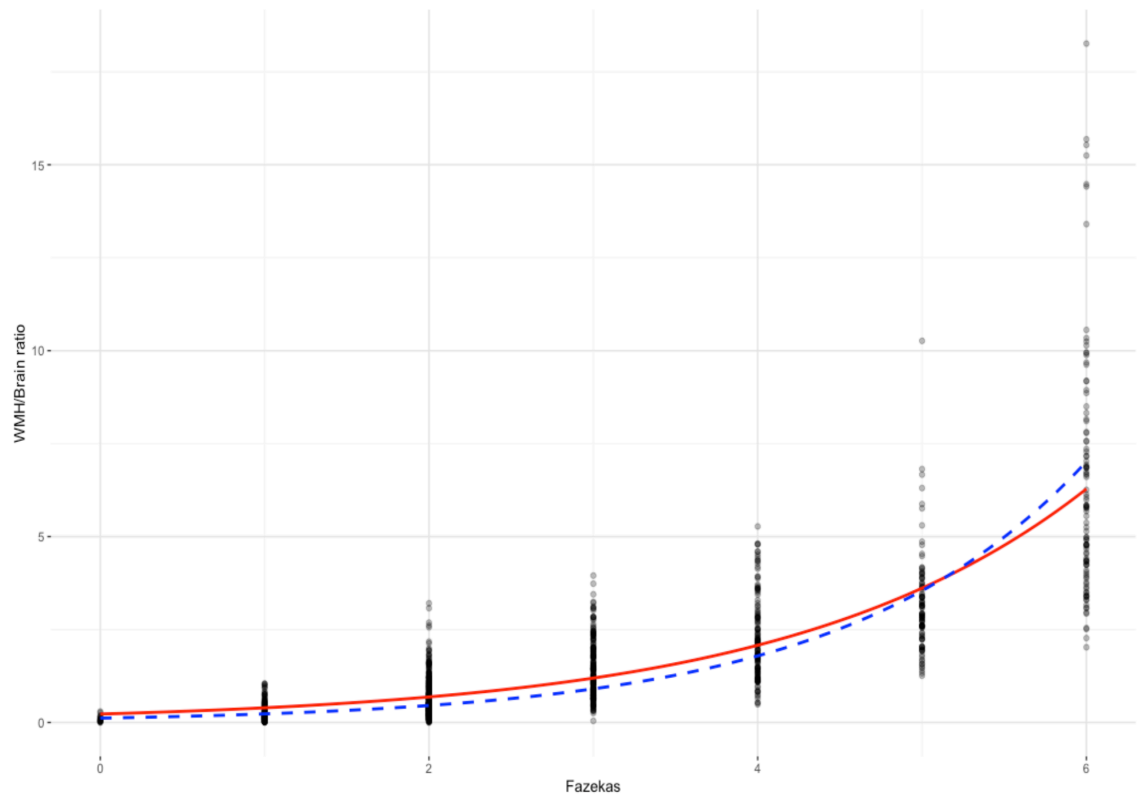

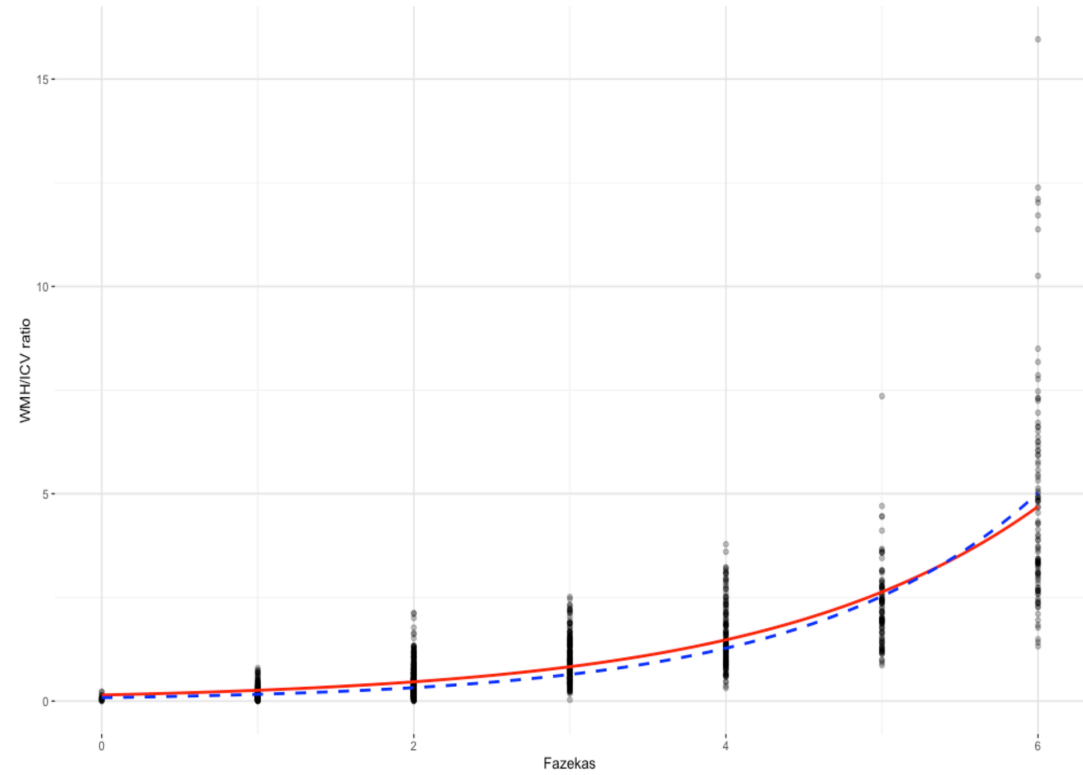

LBC1936

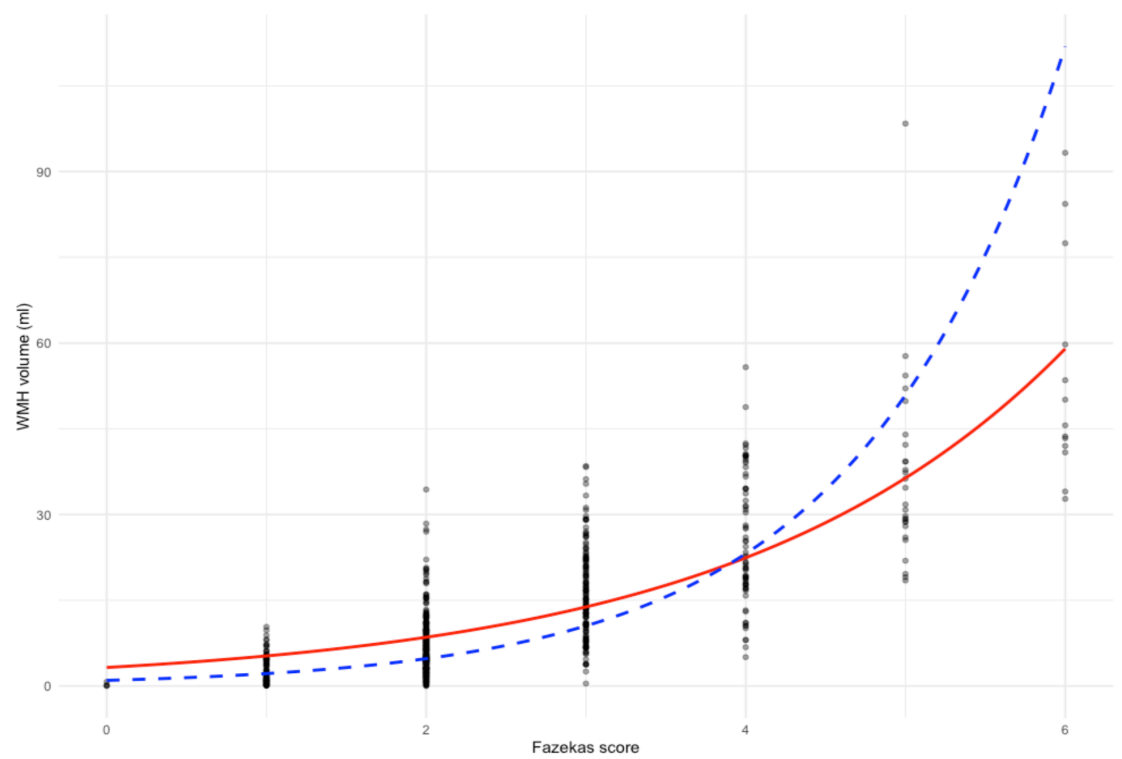

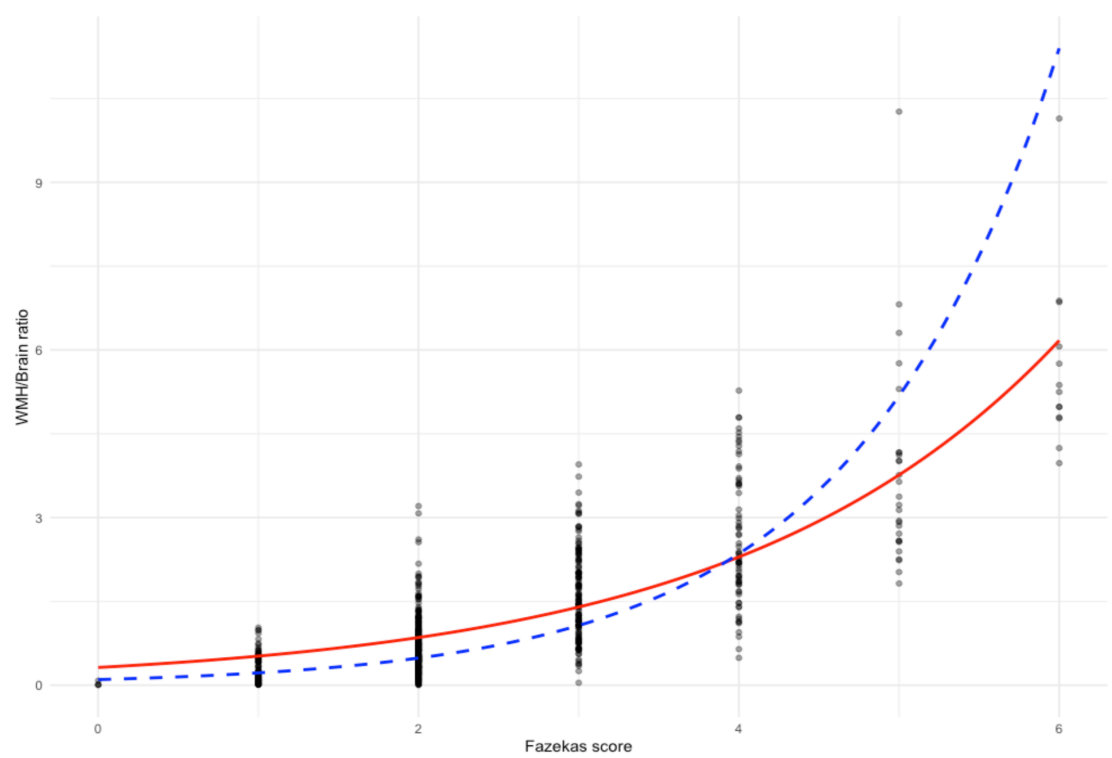

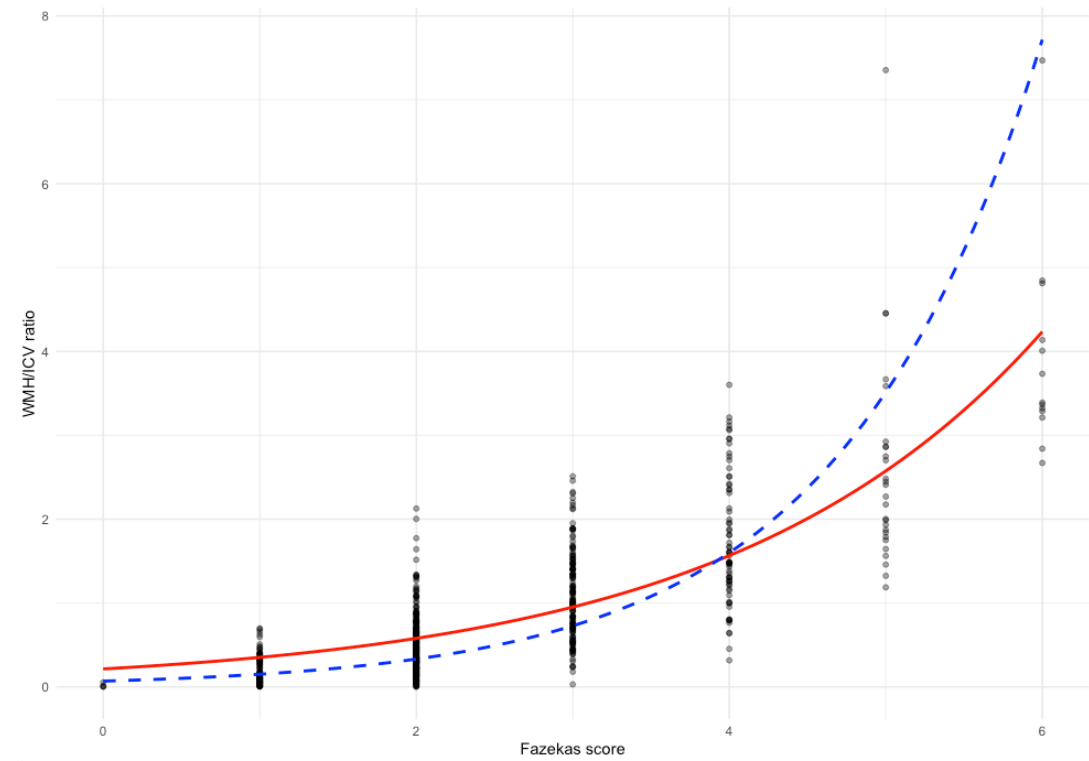

MSS2

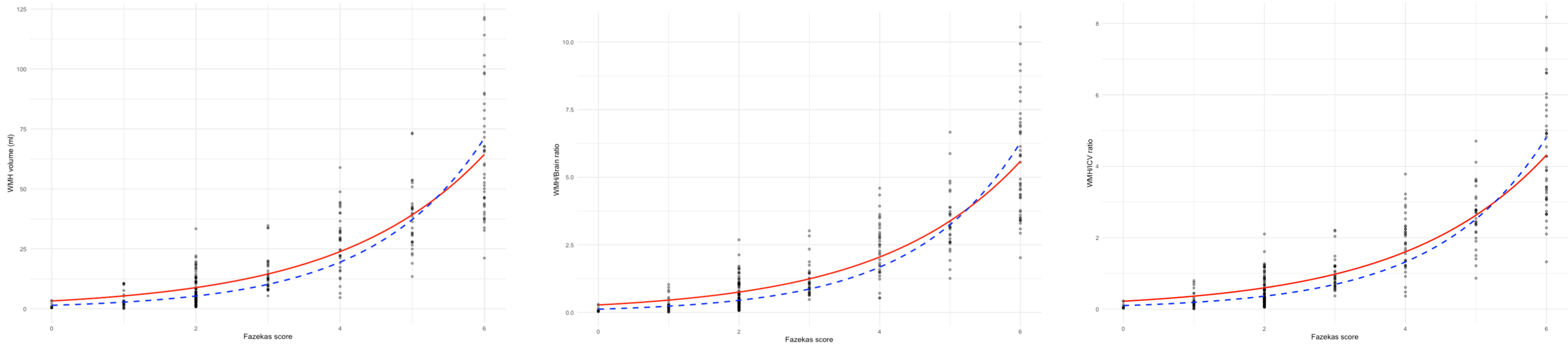

MSS3

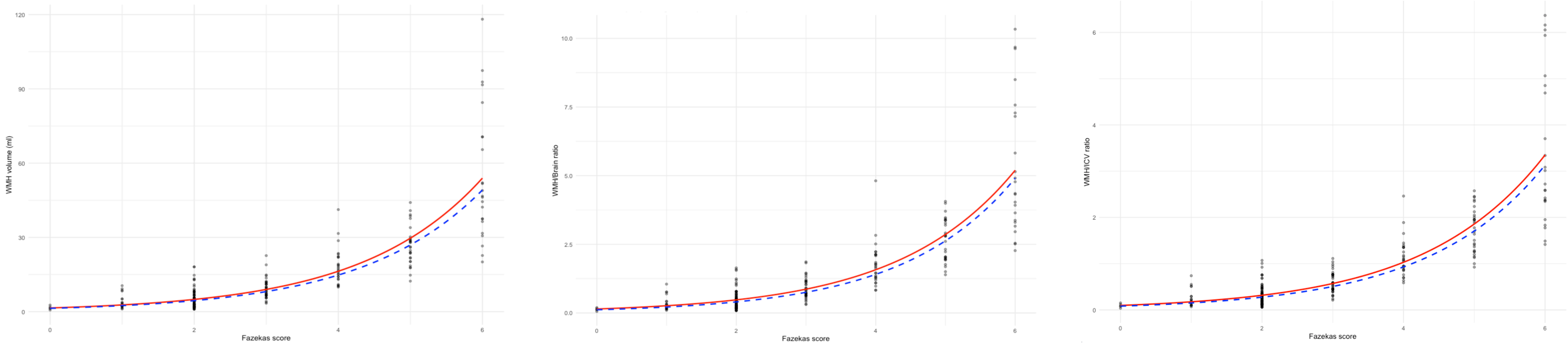

INVESTIGATE

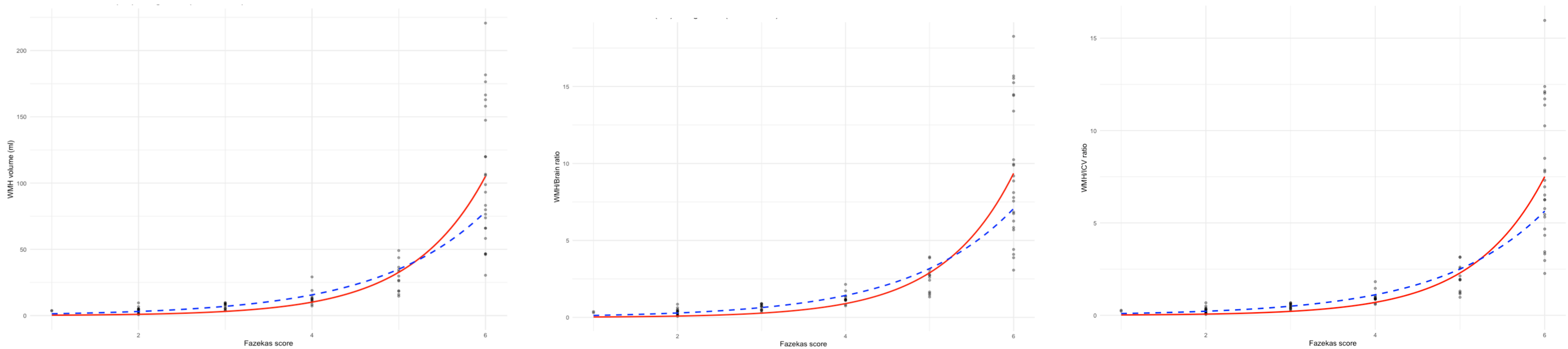

Red lines represent NLS exponential fits and blue dashed lines represent back-transformed log-linear fits. Individual data points are shown for WMH volume, WMH%Brain, and WMH%ICV. Eighteen participants with WMH volume = 0 mL (16 in LBC1936 and 2 in MSS2) were excluded.

STROBE Statement—checklist of items that should be included in reports of observational studies

	Item No	Recommendation	Page No
**Title and abstract**	1	(*a*) Indicate the study's design with a commonly used term in the title or the abstract	4
		(*b*) Provide in the abstract an informative and balanced summary of what was done and what was found	5
**Introduction**			
Background/rationale	2	Explain the scientific background and rationale for the investigation being reported	8
Objectives	3	State specific objectives, including any prespecified hypotheses	8
**Methods**			
Study design	4	Present key elements of study design early in the paper	10-11
Setting	5	Describe the setting, locations, and relevant dates, including periods of recruitment, exposure, follow-up, and data collection	11-13
Participants	6	(*a*) *Cohort study*—Give the eligibility criteria, and the sources and methods of selection of participants. Describe methods of follow-up<br>*Case-control study*—Give the eligibility criteria, and the sources and methods of case ascertainment and control selection. Give the rationale for the choice of cases and controls<br>*Cross-sectional study*—Give the eligibility criteria, and the sources and methods of selection of participants	10
		(*b*) *Cohort study*—For matched studies, give matching criteria and number of exposed and unexposed<br>*Case-control study*—For matched studies, give matching criteria and the number of controls per case	NA
Variables	7	Clearly define all outcomes, exposures, predictors, potential confounders, and effect modifiers. Give diagnostic criteria, if applicable	15
Data sources/ measurement	8*	For each variable of interest, give sources of data and details of methods of assessment (measurement). Describe comparability of assessment methods if there is more than one group	13-15
Bias	9	Describe any efforts to address potential sources of bias	13
Study size	10	Explain how the study size was arrived at	10
Quantitative variables	11	Explain how quantitative variables were handled in the analyses. If applicable, describe which groupings were chosen and why	15
Statistical methods	12	(*a*) Describe all statistical methods, including those used to control for confounding	15-17
		(*b*) Describe any methods used to examine subgroups and interactions	16-17
		(*c*) Explain how missing data were addressed	16
		(*d*) *Cohort study*—If applicable, explain how loss to follow-up was addressed<br>*Case-control study*—If applicable, explain how matching of cases and controls was addressed<br>*Cross-sectional study*—If applicable, describe analytical methods taking account of sampling strategy	NA
		(*e*) Describe any sensitivity analyses	16-17

Continued on next page

**Results**			
Participants	13*	(a) Report numbers of individuals at each stage of study—eg numbers potentially eligible, examined for eligibility, confirmed eligible, included in the study, completing follow-up, and analysed	17
		(b) Give reasons for non-participation at each stage	17
		(c) Consider use of a flow diagram	17
Descriptive data	14*	(a) Give characteristics of study participants (eg demographic, clinical, social) and information on exposures and potential confounders	18
		(b) Indicate number of participants with missing data for each variable of interest	17
		(c) *Cohort study*—Summarise follow-up time (eg, average and total amount)	NA
Outcome data	15*	*Cohort study*—Report numbers of outcome events or summary measures over time	18
		Case-control study—Report numbers in each exposure category, or summary measures of exposure	
		Cross-sectional study—Report numbers of outcome events or summary measures	
Main results	16	(*a*) Give unadjusted estimates and, if applicable, confounder-adjusted estimates and their precision (eg, 95% confidence interval). Make clear which confounders were adjusted for and why they were included	18-20
		(*b*) Report category boundaries when continuous variables were categorized	18-19
		(*c*) If relevant, consider translating estimates of relative risk into absolute risk for a meaningful time period	NA
Other analyses	17	Report other analyses done—eg analyses of subgroups and interactions, and sensitivity analyses	20-21
**Discussion**			
Key results	18	Summarise key results with reference to study objectives	22
Limitations	19	Discuss limitations of the study, taking into account sources of potential bias or imprecision. Discuss both direction and magnitude of any potential bias	26-27
Interpretation	20	Give a cautious overall interpretation of results considering objectives, limitations, multiplicity of analyses, results from similar studies, and other relevant evidence	22-25
Generalisability	21	Discuss the generalisability (external validity) of the study results	26
**Other information**			
Funding	22	Give the source of funding and the role of the funders for the present study and, if applicable, for the original study on which the present article is based	28-30

*Give information separately for cases and controls in case-control studies and, if applicable, for exposed and unexposed groups in cohort and cross-sectional studies.

Note: An Explanation and Elaboration article discusses each checklist item and gives methodological background and published examples of transparent reporting. The STROBE checklist is best used in conjunction with this article (freely available on the Web sites of PLoS Medicine at http://www.plosmedicine.org/, Annals of Internal Medicine at http://www.annals.org/, and Epidemiology at http://www.epidem.com/). Information on the STROBE Initiative is available at www.strobe-statement.org.
